# Computing the pH-Dependent Thermodynamics of the Allostery between Dimerization and Palmitate Binding in β‑Lactoglobulin

**DOI:** 10.1021/acs.jpcb.5c01119

**Published:** 2025-05-23

**Authors:** Lucie da Rocha, Sara R. R. Campos, António M. Baptista

**Affiliations:** Instituto de Tecnologia Química e Biológica António Xavier, 98819 Universidade Nova de Lisboa, Av. da República, Oeiras 2780-157, Portugal

## Abstract

The study of pH-dependent allosteric processes presents a significant challenge, both experimentally and computationally. In this work, we apply the constant-pH molecular dynamics method to explore an interesting case of allostery involving protein–ligand binding and dimerization. As a model system, we use β-lactoglobulin (BLG), a small protein from bovine milk known to dimerize and bind palmitic acid in a hydrophobic pocket—both processes sensitive to pH. This study focuses on the holo form of BLG, and, when combined with our previous study of the apo form (da Rocha et al. *J. Chem. Theory Comput.* **2022** 18, 1982), completes the thermodynamic cycle of the allosteric process. The corresponding pH-dependent free energy profiles are obtained through the use of a thermodynamic linkage relation, avoiding the need of performing heavy computational calculations. Dimerization is found to be more favorable near the isoionic point, as observed in the apo form. Palmitate binding is found to be more favorable around pH 6–7, a biologically relevant pH range at which the gate covering the binding site is known to open. A pH-dependent measure of allosteric coupling is computed, showing that ligand binding and dimerization exhibit an antagonist relationship within the studied pH range of 3–8, with binding destabilizing dimerization and vice versa.

## Graphical abstract

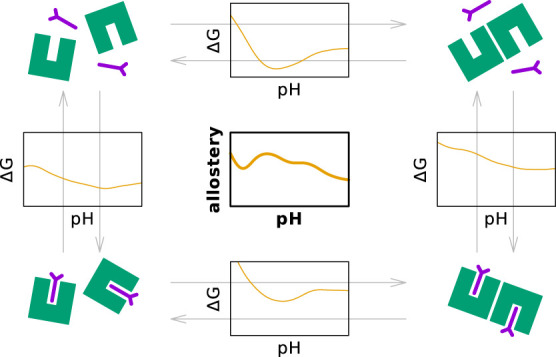

## 1 Introduction

Allosteric regulation provides a major mechanism for modulating biochemical function.^1,2^ The concept was introduced to describe the mechanism by which the binding affinity of a ligand can be affected by the binding, at a different site, of a so-called allosteric effector that induces a conformational transition.^3,4^ This transition can be more generally regarded as a shift of relative populations of distinct structural ensembles^5^ and might even consist of a simple change of fluctuations.^6^ The concept of allostery is sometimes used to refer to any ligand-induced conformational change,^1^ even if a single binding site is involved, but the usefulness of such broadening has been questioned^7^ and it is not adopted in the present article.

An interesting situation occurs when protein dimerization (or, more generally, oligomerization) is affected by the binding of a small ligand away from the protein–protein interface. In this case the protein itself can be regarded as a second type of ligand, with its binding leading to the formation of precisely the type of identical-units oligomer often found in classic allosteric systems (e.g., hemoglobin).^4^ Furthermore, the usual pH dependence of biomolecular processes implies that acid–base equilibrium will typically affect both the dimerization and the binding of the small ligand, meaning that the protons provided by the solution may be seen as a further type of allosteric effector. Thus, we may regard such a system as subjected to a complex allosteric process in which dimerization, binding of a (possibly titratable) small ligand, and acid–base equilibrium are all coupled. In a more traditional view, pH can be simply regarded as an external parameter that modulates the dimerization–binding allostery.

The identification of the molecular-level mechanisms of allostery is a challenging task, to which computational methods have given a valuable contribution. Traditionally, these methods have focused on identifying networks of short-range residue interactions, whose dynamics might explain the transmission of ligand-induced changes between allosteric sites.^8–11^ However, the consideration of acid–base equilibrium opens the possibility for the allosteric changes to be mediated by long-range electrostatic interactions, and rigid-structure studies based on Poisson–Boltzmann (PB) models have indeed identified many interesting cases in which protonation and ligand binding (including dimerization) affect each other.^12–20^ The effects of both dynamics and protonation can be studied using constant-pH MD (CpHMD) methods,^21–26^ which have been used to study the effect of pH on ligand binding^27–32^ and dimerization.^33–35^ Some of the studied receptors are synthetic and have no titratable sites for binding protons.^27–29^ The others consist of proteins that bind nontitratable^30,31^ or titratable^32^ small ligands, or are involved in dimerization,^33–35^ and which might be regarded as allosteric since, in addition to the site for binding the small ligand or dimer partner, they have titratable sites for protons. However, the ubiquitous acid–base equilibrium of proteins tends to be regarded as a “background” effect that, though being able to modulate allostery, is not traditionally regarded as part of it. In this study, we extend the approach of previous CpHMD studies to address a case of traditional allostery, namely the one described above in which protein dimerization is coupled with ligand binding of a titratable ligand, while being modulated by acid–base equilibrium. In particular, we intend to derive a convenient pH-dependent measure of the dimerization–binding allostery.

The system studied here is β-lactoglobulin (BLG), a small β-barrel protein consisting of 162 amino acids, which is the major component of the serum of bovine milk and can cause an allergic reaction in humans.^36^ BLG exhibits a pH-dependent dimerization equilibrium, with the dimer being more favored near its isoionic point^35,37–43^ (∼5.2 ^44,45^ ), possibly experiencing a small structural transition around pH 4.^35^ BLG isolated from bovine milk contains various bound fatty acids, the most abundant being palmitate (PLM),^46^ and can also bind other small hydrophobic ligands in vitro.^47^ This suggests not only a possible biological role as a transporter but also the potential to be exploited as a carrier of bioactive compounds.^48^ Most ligands seem to bind to BLG’s β-barrel cavity through a pH-regulated mechanism involving the movement of the EF loop located at the entrance of the hydrophobic cavity.^49^ This movement probably corresponds to the so-called Tanford transition, a structural change occurring around pH 7–8.^50^ The EF loop can act as a pH-triggered gate that assumes a closed conformation at lower pH, inhibiting ligand binding, and opens as pH is increased, facilitating ligand binding.^49^ Indeed, binding of palmitate^51,52^ and other fatty acids^52^ to BLG is found to increase as one crosses the pH of the Tanford transition. Furthermore, the crystallographic structure of the BLG–PLM complex shows PLM with its tail deeply inserted into the hydrophobic cavity and its carboxyl group facing the solvent at the cavity entrance, closely interacting with Lys-60 and Lys-69.^53,54^

The dimerization of BLG and its binding of palmitate correspond to a joint equilibrium involving five different protein forms: apo monomer (M), holo monomer (ML), apo dimer (D), and the singly- and doubly occupied holo dimer (DL and DL_2_). The full characterization of this equilibrium thus requires four independent equilibrium constants or reaction free energies. Since we have not performed simulations of the DL form, the transformations involving this form are not addressed here, leaving us with the single (though incomplete) thermodynamic cycle shown in Figure 1. Since the free energies depend on pH, this cycle also expresses the relation between the four pH-dependent free energy profiles. One of those profiles, Δ*G*_dim_^apo^(pH), has already been determined in our study of the dimerization of the apo form, meaning that we just need to determine two more independent ones, say Δ*G*_bind_^M^(pH) and Δ*G*_bind_^D^(pH); the profile Δ*G*_dim_^holo^(pH) is then given by

1 $$\Delta G_{\dim}^{holo}=\Delta G_{\dim}^{apo}+\Delta G_{bind}^{D}-2\Delta G_{bind}^{M}$$

**Figure 1:**
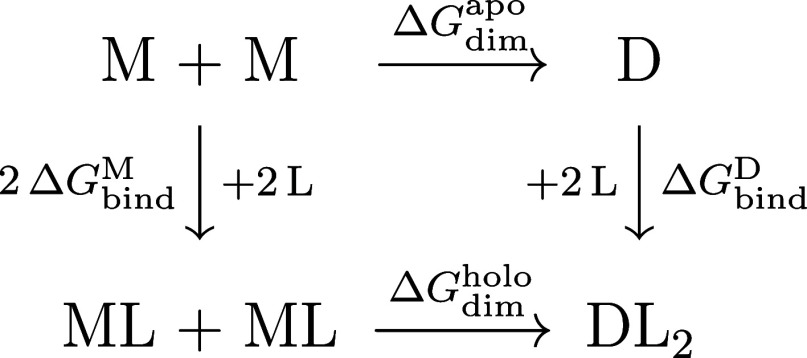
Thermodynamic cycle of the allosteric process involving the dimerization of BLG and its binding of palmitate.

In the present work we perform CpHMD simulations of the holo forms of the BLG monomer and dimer, required to complete the cycle in Figure 1. The free energies are computed with a differential linkage relation^1,55^ integrated using a thermodynamically based spline,^35^ with the absolute values being obtained by reference to experimental data.^56^ The data for the apo forms are taken from our previous study.^35^ We then compute a pH-dependent measure of the allosteric coupling between palmitate binding and dimerization in BLG.

## 2 Theory and Methods

### 2.1 Structural Models

The structural models used were the holo forms of the monomer and dimer of bovine β-lactoglobulin variant A, with the binding pocket occupied with a palmitate molecule. The holo monomer was chosen from the protein data bank (PDB code 1b0o^53^). Since this structure corresponds to variant B, we performed the mutations Gly64Asp and Val118Ala to convert it to variant A.^36^ The dimer was created by generating symmetry partners within 20 Å using PyMOL^57^ and, subsequently, choosing the proper partner after comparing it with the existing, although incomplete, apo dimer PDB structure (PDB code 1beb^58^). For convenience of data processing, palmitate is shown in some figures as residue number 180, as originally assigned in 1b0o.

The monomer and dimer were solvated with a total of 9604 and 36,292 water molecules, respectively, in a rhombic dodecahedral box. Sodium and chloride ions were introduced to replace some of the solvent molecules, to achieve an approximately neutral simulation system at an ionic strength of 0.1 M, using a protocol previously described.^59^

### 2.2 Molecular Mechanics and Molecular Dynamics Settings

The MM/MD simulations were performed using the GROMOS 54A7 force field^60^ with the SPC water model,^61^ and an in-house^62^ modified GROMACS package version 2018.3.^63^ To maintain structural stability, constraints were applied to the protein bonds and water bonds and angles, using the LINCS^64^ and SETTLE^65^ algorithms, respectively. Nonbonded interactions were treated with a Verlet cutoff of 1.4 Å, with neighbor lists being updated every simulation step.^63^ Electrostatic interactions were treated using the generalized reaction field method,^66^ using a dielectric constant of 54 ^67^ and an ionic strength of 0.1 M.The temperature was kept at 300 K by applying the v-rescale coupling bath,^68^ with a relaxation time of 0.1 ps, and the pressure at 1 atm, using a Parrinello–Rahman coupling bath^69^ with a relaxation time of 0.5 ps. The integration time step was 0.002 ps, using the leapfrog algorithm.

Before performing the constant-pH MD simulations, the system was subjected to energy minimization and structural relaxation, which is particularly relevant due to the mutations performed in the structure. First, we performed a two-step energy minimization restraining all non-hydrogen atoms with a force constant of 1000 kJ mol^–1^ nm^–2^ using a steepest descent algorithm; this was followed by ∼10,000 steps with no restraints. Next, we carried out three sequential MD simulations of standard MD. The first was a 50 ps simulation in the *NVT* ensemble, with all non-hydrogen atoms restrained. Subsequently, we performed a structural relaxation where only the C^α^ atoms were restrained. Finally, we ended with a 100 ps NPT simulation with all the C^α^ atoms restrained. The position restraint force constant was the same for all simulations. Conformations were saved every 10 ps.

### 2.3 Constant-pH MD Simulations

Constant-pH MD simulations were performed using the stochastic titration method developed by Baptista and co-workers.^21,63^ This approach involves performing an MM/MD simulation, and doing periodic interruptions to update the protonation states through Poisson–Boltzmann (PB) and Monte Carlo (MC) calculations, at a specific pH. This ensures a proper sampling of both conformational and protonation states.^21^ All constant-pH simulations were performed for 200 ns at pH values 3, 4, 5, 6, 7, and 8. For both monomer and dimer, 8 replicates were performed at each pH, started with different initial velocities, which amounts to 1.6 μs per pH value and a total of 19.2 μs of simulation time. The two chains remained associated in all dimer simulations.

PB/MC calculations were performed every 10 ps, followed by 0.2 ps MD step of solvent relaxation^21^ with the updated protonation states. Proton tautomerism was applied to all sites titratable within the pH range used.^70,71^ The free Cys121 was kept neutral since it exhibited a p*K*_a_ ≥ 15 in preliminary simulations, and remained deeply buried within a hydrophobic region during the subsequent CpHMD simulations, as previously observed in our study of the apo form.^35^ To reduce computer cost, a reduced titration approach^63^ was used, where a site exclusion list was updated every 50 cycles of CpHMD, with a threshold of 0.999 protonation state frequency.

The PB equation was solved using the program MEAD, version 2.2.9.^72^ In practice, this is obtained using the finite difference method with a two-step focusing approach with grid spacings of 1.0 and 0.25 Å. The atomic charges and radii were obtained from the GROMOS 54A7 force field as described in ref 71. The molecular surface was obtained using a solvent probe of radius 1.4 Å and a Stern layer of 2.0 Å, whereas the dielectric constant was set to 2 for the molecular interior and 80 for the solvent. The temperature was set to 300 K and the ionic strength was set to 0.1 M. The preparation of all the necessary files for the PB calculations with tautomers was done using the in-house package meadTools (version 2.2).^62,70^ The sampling of the protonation states was done using the MC method, implemented in the PETIT program, version 1.6.^62,70,73^ 10^5^ MC cycles were performed, with each cycle consisting of a stochastic selection of states according to the Metropolis rule^74^ for all individual sites and for pairs of sites with a coupling above 2.0 p*K*_a_ units.^73^ The p*K*_a_ values of the amino acid model compound fragments^75^ for the GROMOS 54A7 force field where the ones previously obtained.^76^

### 2.4 Palmitate Parametrization

The palmitate molecule was parametrized by analogy with similar molecular moieties of the GROMOS 54A7.^60^ The hydrophobic tail was assigned the same parameters as a typical aliphatic chain (e.g., as in DPPC), and the carboxyl head was assigned the same parameters used for the side chain of glutamic acid. This head was used as the model compound fragment^75^ in a series of 100 ns CpHMD simulations of palmitate in water conducted within a pH range of 3–7, using 3 replicates for each pH value. The p*K*_a_ of this model compound was initially set to 4.19, like for the Glu model compound fragment.^76^ After performing the simulations, the obtained average protonations were pH-shifted to match the titration curve corresponding to the experimental p*K*_a_ of palmitic acid (5.0, as extrapolated from other fatty acids^2^). This shift was then added to the initial p*K*_a_ of the model compound of palmitic acid, thus assigning it a final value of 4.99.

### 2.5 Calculation of Relative Free Energies from Linkage Relations

Protein protonation curves are easily obtained using the average protonations at the different simulated pH values. These protonation curves can then be used to obtain a pH-dependent relative reaction free energy, by taking advantage of a linkage relation.^1,55^ Its application to the BLG dimerization reaction was already described for the apo form^35^ and can be easily extended to the binding of palmitate. For example, the relative free energy of the binding of palmitate (L) to the monomer (M) is given by

2 $$\Delta \Delta G_{bind}^{M}\left(pH\right)=\ln\left(10\right)RT\int_{{pH}_{ref}}^{pH}\left({\mathrm{n̅}}^{ML}-\left({\mathrm{n̅}}^{M}+{\mathrm{n̅}}^{L}\right)\right)d{pH}^{\prime }$$

where ${\mathrm{n̅}}^{X}$ is the average protonation of species X and pH_ref_ is a reference pH value. The contribution of each site *i* to this relative change is

3 $$\Delta \Delta G_{bind,i}^{M}\left(pH\right)=\ln\left(10\right)RT\int_{{pH}_{ref}}^{pH}\left({\overset{®}{n_{i}\;}}^{ML}-{\overset{®}{n_{i}\;}}^{M}\right)d{pH}^{\prime }$$

where ${\overset{®}{n_{i}\;}}^{X}$ is the average protonation of site *i* in species X; in the case of the palmitate contribution, ${\overset{®}{n_{i}\;}}^{M}$ is replaced by ${\mathrm{n̅}}^{L}$ . Analogous relations hold for the dimer, involving the binding of two palmitate molecules. Since the pH-dependent free energy profiles thus obtained are defined up to a constant, their vertical placement is here assigned by reference to experimental data, as done in the dimerization study of the BLG apo form.^35^ The average protonations of the holo forms were obtained from the simulations done in the present work, that of palmitate was derived from its experimental p*K*_a_ of 5.0, and those of the apo form were taken from our previous study.^35^

For this approach to be reliable, a sound method for calculating the integrals of the obtained protonation curves must be used, considering the few and discontinuous pH values at which the CpHMD simulations were run. So, we use a spline-based integration method^35^ to solve this integral, based on the fact that the slopes of the total and individual protonation curves can be computed from, respectively, the (co)­variances var­(*n*) and cov­(*n*_*i*_, *n*) directly obtained from the CpHMD simulations, allowing a more accurate analysis. Additionally, we compare the results with the more approximate Hill-based integration method.^33^ See ref 35 for more details.

### 2.6 Principal Component Analysis

Principal component analysis (PCA)^77^ was used to obtain a pH-dependent structural characterization of the bound palmitate molecule. PCA operations were performed using the Python scikit-learn package,^78^ after carefully selecting the structural fitting of the input structures and the coordinates to be PCA-transformed.

The PCA of PLM was performed using the equilibrated frames obtained at different pH values. Each BLG chain (excluding palmitate) had its backbone atoms fitted to the crystallographic structure, and the resulting Cartesian coordinates of all PLM atoms were PCA-transformed. This approach allows a characterization of both the position and conformation of PLM. The first two PCs captured always more than 68% of the total variance.

Energy landscapes were obtained in the space of the first two PCs, using the program getdensity of the package LandscapeTools.^62,79^ The probability density was computed on a grid using a Gaussian kernel density estimator with a bandwidth of σ­(4/3*N*)^1/5^, where σ is the standard deviation of the *N* sampled points.^79,80^ The mesh size used was 0.5 Å. The corresponding free energy surface was calculated according to

4 $$E\left(\mathrm{r⃗}\right)=-RT\ln\frac{P\left(\mathrm{r⃗}\right)}{P_{\max}}$$

where *r⃗* is the coordinate in the 2-dimensional space and *P*_max_ is the maximum of the probability density function, P(r⃗) .

Energy landscape basins were determined using the program getbasins of the LandscapeTools package^62,79^ with a cutoff of 2 *RT*.

### 2.7 Other Analyses

Trajectory processing accounting for periodic boundary conditions was done using FixBox,^62,81^ and standard analyses were conducted on the last 170 ns of each simulation using the GROMACS package^82^ and in-house tools. An equilibration time of 30 ns was decided based on the observation of the temporal evolution of several properties.

The titration curves were obtained by averaging the occupancy states of each titratable site at various pH values. The p*K*_a_ and Hill coefficient *h* for each titratable site were obtained by fitting to the average protonations a Hill curve

5 $$f\left(pH\right)={\left[1+10^{h\left(pH-pK_{a}\right)}\right]}^{-1}$$

These fits were performed using the Marquardt–Levenberg nonlinear least-squares algorithm^83^ implemented in gnuplot.^84^

The statistical uncertainties of the analyses presented herein were determined either as the standard deviation over replicate averages, or, in the case of protonation-derived quantities, with the bootstrap method described in ref 85, using 1000 resamples. The bootstrap uncertainties were expressed either as ±1 standard deviation of the averages of the bootstrap resamples or, in the case of the average protonations, as a 68% confidence interval of those resamples.

The probability densities of the sodium and chloride ions around the protein were calculated with the program LandscapeTools,^62,79^ using a triangular kernel with a bandwidth of 2 Å. The positions of sodium and chloride ions were determined after the protein was fitted to a central structure^79^ and their probability densities were calculated on a grid of mesh 1 Å and converted to concentrations in mM.

The coupling between the protonation of pairs of titratable sites was measured using the Pearson correlation coefficient,^86^ which can detect direct interactions between pairs of proton-binding sites and also indirect effects mediated through other sites.^73^

The protein solvent-accessible surface area (SASA) was computed with the GROMACS tool gmx sasa using a rolling probe with a radius of 0.14 nm. The contact surface area between the two dimer partners was calculated as

6 $$\left({SASA}_{A}+{SASA}_{B}-{SASA}_{dimer}\right)/2$$

where *A* and *B* represent each individual dimer partner.

Histograms were computed using a Gaussian kernel estimate with a bandwidth of σ­(4/3*N*)^1/5^, where σ is the standard deviation of the *N* sampled points, as implemented in gnuplot,^80,84^ and normalized using 1/*N*.

All molecular representations were done using the PyMOL software.^57^

## 3 Results and Discussion

### 3.1 Protonation Curves

The global protonation curves for both the monomer and dimer forms of holo BLG are depicted in Figure 2, with a solid line. Additionally, the protonation of the apo form (previously obtained^35^) is represented by a dashed line. These results closely resemble the ones of the titration of the apo form, as expected, since the holo form only has an additional charge that is contributed by the palmitate. In particular, the isoionic point (pI) obtained for the holo form is 5.0, very close to the apo one (5.1 from simulations^35^ and 5.2–5.4 from experiments^44,87^ ). However, upon analyzing the titration of individual residues (Figure S1 in Supporting Information), it becomes evident that while most exhibit similar behavior to that of the apo form (Figure S1 in ref 35), some display differences, which are associated with the residue-specific contributions to the pH-sensitivity of the binding free energy (see next section). The respective p*K*_a_ values are also shown in Table S1 in Supporting Information.

**Figure 2:**
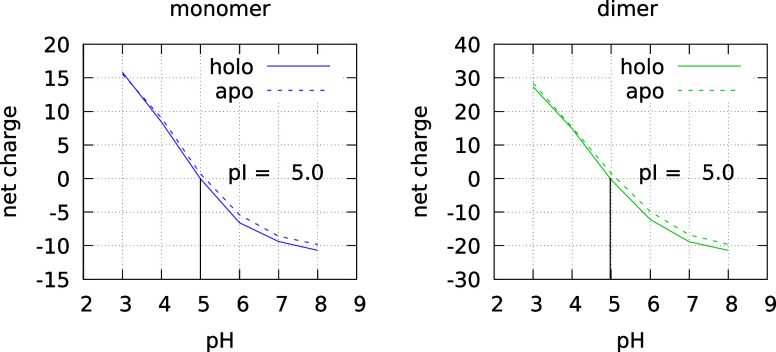
Mean protein charge as a function of pH for the monomer (purple) and dimer (green), for the holo (solid line) and apo^35^ (dashed line) forms; the statistical uncertainty is always lower than half a charge unit. The pI is also shown.

### 3.2 Ligand Binding Free Energy

As pointed in Section 2.5, the pH-dependent free energy profiles obtained from the linkage relation are defined up to a constant. This constant can be obtained through experimental data^35^ or computed from absolute free energy calculations.^27,32^

In order to obtain the absolute value of the free energy of PLM binding, we have decided to use the experimental values in the work of Wang et al.,^56^ since their experimental conditions are the only ones that match those used in our previous work on the apo form, as well as the current study on the holo form, besides having tested different BLG concentrations. More experimental studies on PLM binding free energy were performed^51,52,54,56,88–93^ but they were unsuitable for a reasonable comparison, as explained in more detail in the Supporting Information. In this study, even though they did not reach concentrations where BLG was exclusively in the monomeric or dimeric form, we decided to consider the monomeric and dimeric forms as approximately represented by the reported BLG concentrations of respectively 1 μM and 200 μM, thus assigning Δ*G*_bind_^M^ = −8.3 kcal/mol and Δ*G*_bind_^D^ = −14.5 kcal/mol, obtained from the single-chain binding constants reported for those concentrations.^56^ However, since these concentrations do not still correspond to the fully monomeric and fully dimeric forms, the actual difference between Δ*G*_bind_^M^ and Δ*G*_bind_^D^ is expected to be slightly greater.

The ligand binding free energies of the monomer and dimer are presented in Figure 3, using both the spline- and Hill-based methods. The curves were vertically fitted to the respective experimental values reported in the work of Wang et al.,^56^ and both have a favorable binding free energy. In both the monomer and dimer, the two methods exhibit different shapes but have a similar overall trend. In the monomer, there is a slight increase between pH 3 and 4, followed by a progressive decrease along the pH range until it reaches a minimum around pH 6.5. This indicates that the binding of PLM is more favorable between pH 6 and 7, close to the pH range where the Tanford transition occurs, which is interesting to note. In the dimer, the binding free energy decreases until it reaches pH 7, in both the splines and the Hill curve approaches, with a slight increase around pH 7.5 in the spline curve. In both forms, there is a binding increase from the acidic to the neutral region, with the binding in the monomer being more favorable; this is a direct consequence of the fit to the experimental data at pH 7 obtained by Wang et al.^56^ In fact, as noted above, the difference is expected to be even slightly greater.

**Figure 3:**
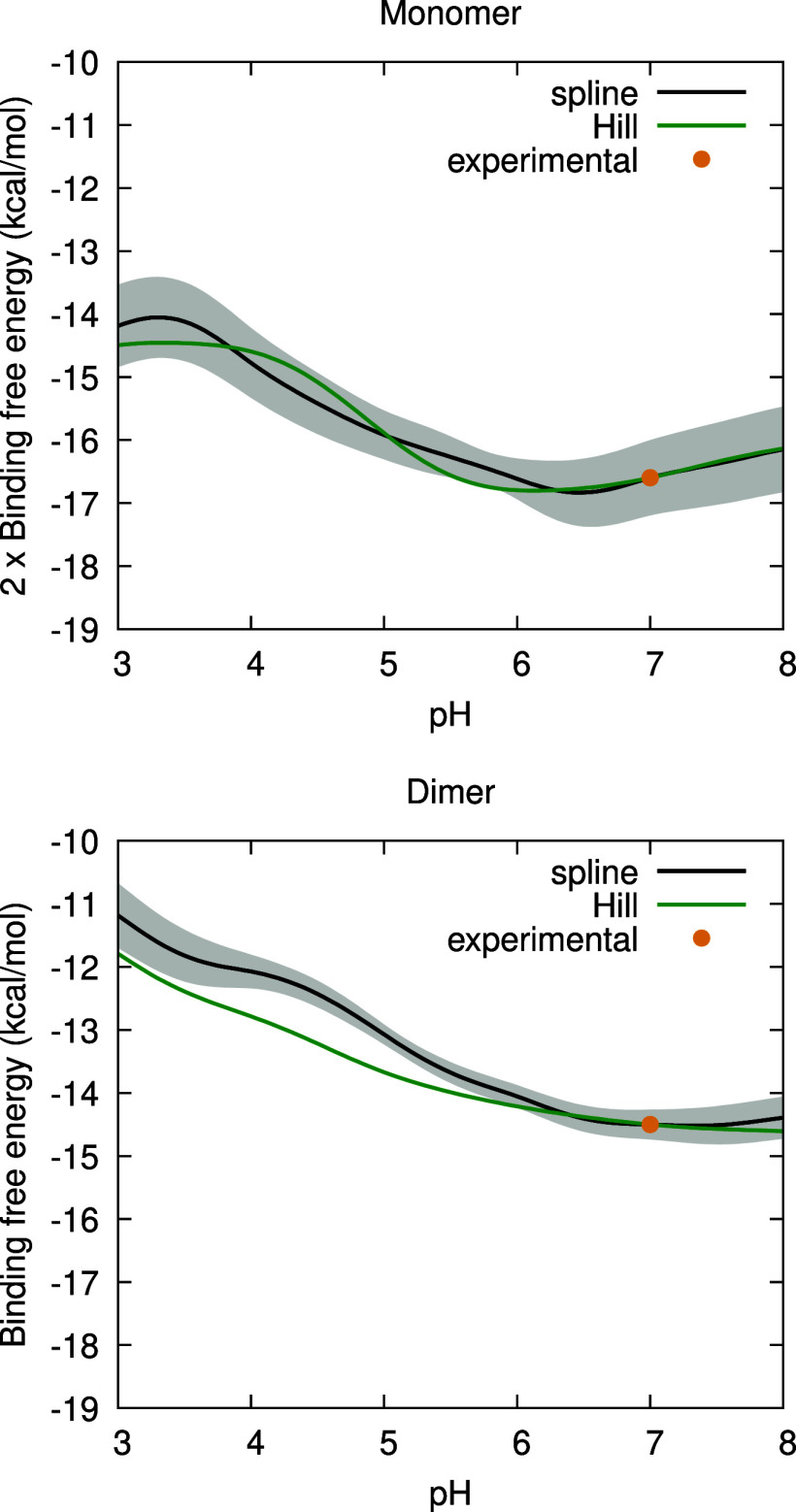
Binding free energy as a function of pH, calculated with the splines-based (black line) and the Hill-based (green line) methods. The gray shadow area delimits the error bounds obtained using a bootstrap method, explained in ref 85. The yellow dots correspond to the experimental points obtained at pH 7 in ref 56 (Δ*G*_bind_^M^ = −8.3 ± 0.003 kcal/mol and Δ*G*_bind_^D^ = −14.5 ± 0.008 kcal/mol).

These results indicate that the binding of PLM is more favorable close to the pH range where the Tanford transition occurs (7–8).^50^ This is in agreement with previous experiments,^52^ likely reflecting the fact that binding requires the opening of the EF-loop gate in the apo form, which takes place in this pH range.^49^ Biologically, this may be related with the possible role of BLG in enhancing the activity of ruminant pregastric lipases through the sequestering of inhibiting fatty acids, thus assisting in the digestion of milk lipids during the neonatal period.^88^ Since this takes place before the acidic gastric phase, these lipases act essentially at the milk pH, which for ruminants typically ranges from 6.5 to 6.9.^94^ Thus, the pH range of optimal binding observed in Figure 3 may directly reflect an evolutionary optimization of the action of pregastric lipases.

The pH profiles of the site-specific contributions to the binding free energies were analyzed (Figures 4 and 5) and the residues that contribute the most to their pH-dependency were identified in the protein structure (Figure 6). For the monomer, these were Asp-53, Glu-89, Glu-108, Glu-112, CTIle-162 and PLM. Residues Glu-89, Glu-108 and Glu-112 are located in loops near the entrance of the hydrophobic pocket, but they do not exhibit direct interactions with PLM through hydrogen bonds or ion-pairs. In the case of Glu-108 and Glu-112, that would require extensive structural rearrangements which are not observed (see Figure 6), and Glu-89 is never observed nearer than 7 Å from PLM. It should be noted that the protonation states selected during the simulations derive exclusively from the periodical PB calculations, meaning that the charge differences originating the profiles result from differences in the shape of the low-dielectric region and the charge distribution within. These are mainly affected by changes in the PLM binding pose in the holo form (which is affected by pH; see Section 3.3) and also, not to be forgotten, changes in the conformation of the EF loop in the apo form.^35^ A more detailed analysis is made difficult by the diversity of effects at play: conformation, protonation and solvation in both the apo and holo forms at different pH values.

**Figure 4:**
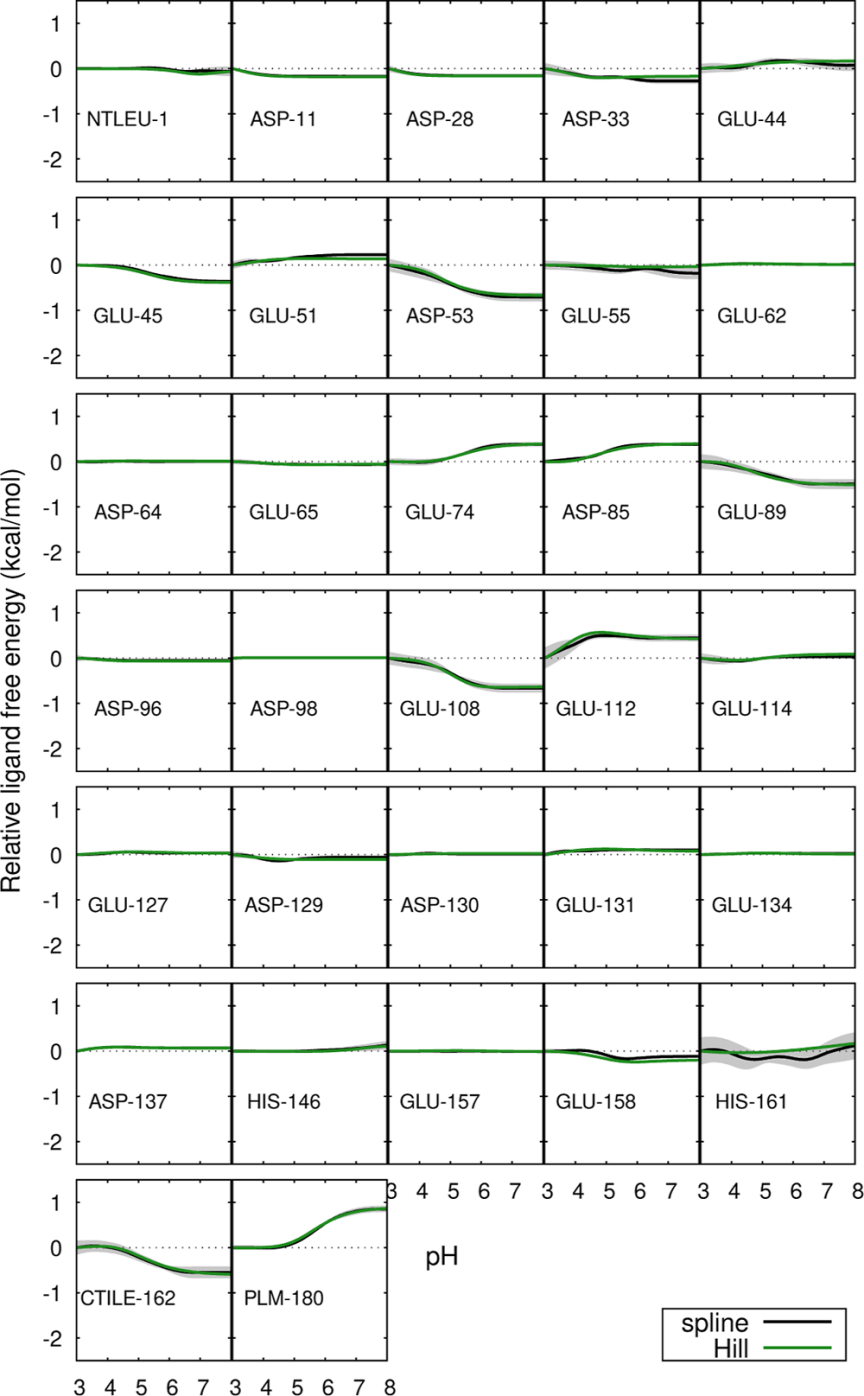
Site-specific contributions for the ligand binding free energy of the monomer relative to pH 3, as a function of pH. For further details, see the caption of Figure 3.

**Figure 5:**
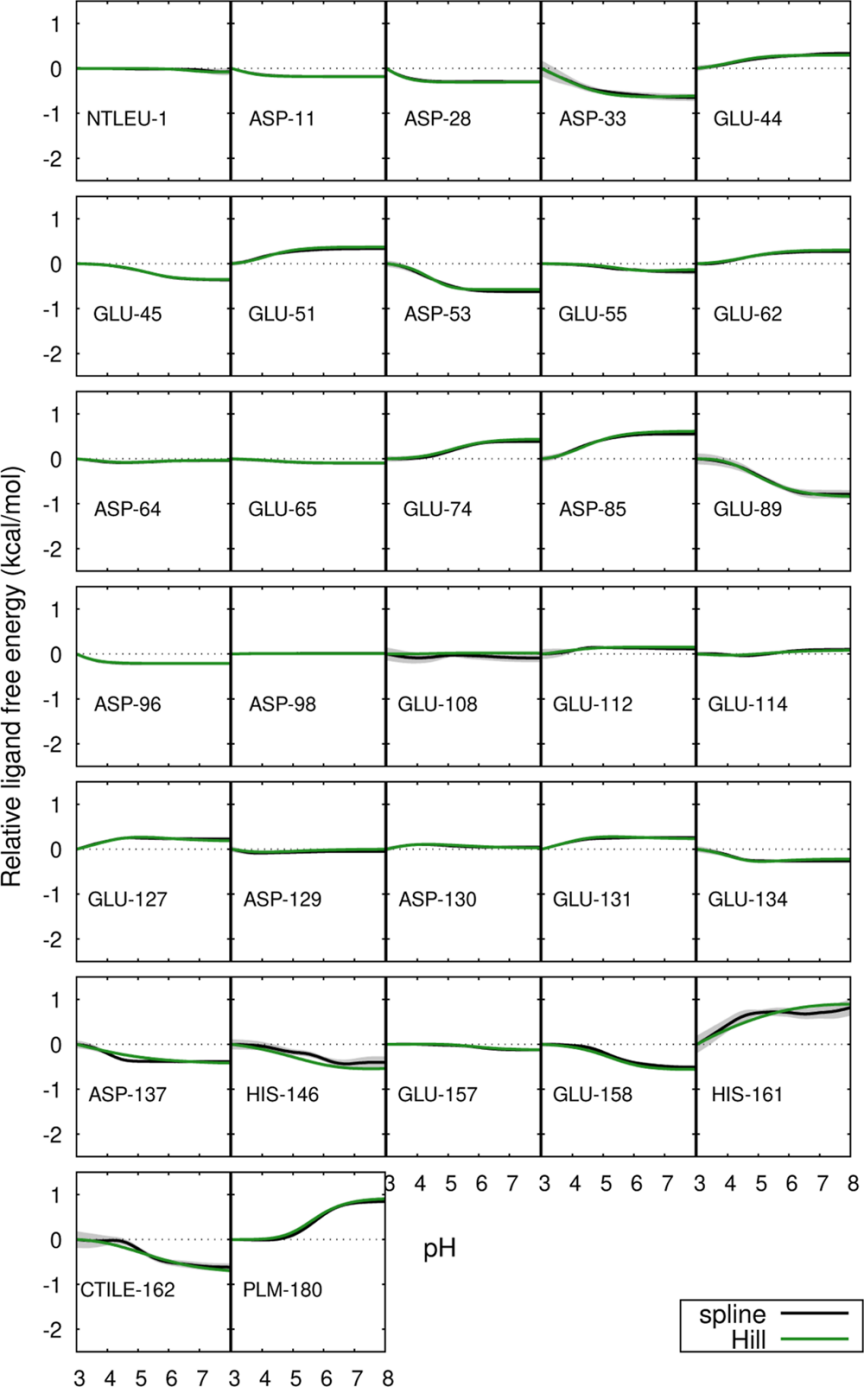
Site-specific contributions for the ligand binding free energy of the dimer relative to pH 3, as a function of pH. For further details, see the caption of Figure 3.

**Figure 6:**
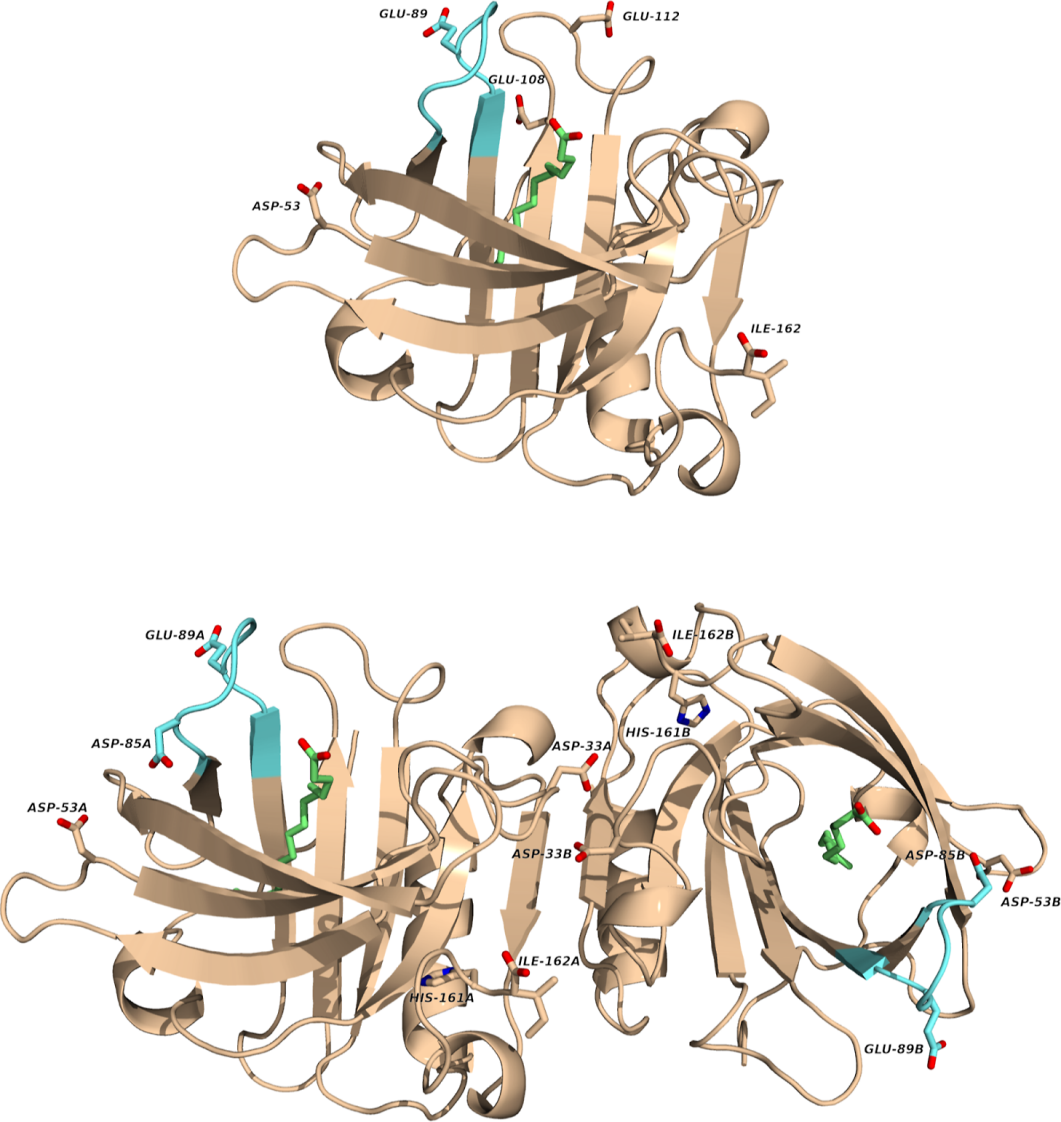
Monomer and dimer representations using sticks to highlight the residues that contribute the most to the pH-dependency of the PLM binding free energy. The palmitate molecules are shown in green, while the EF loops are represented in cyan.

For the dimer, the residues that contribute the most to the pH-dependency of the binding free energy are Asp-33, Asp-53, Asp-85, Glu-89, His-161, CTIle-162 and PLM. Some of these residues are near the interface of the dimer, while others are in the EF loop near the entrance of the hydrophobic pocket, and none of them exhibit direct interactions with PLM. As in the case of the monomer, these profiles ultimately derive from the PB calculations. Besides the effects referred for the monomer (PLM binding pose and EF loop conformation), the relative orientation of the dimer partners must also be considered. As explained in Section 3.6, this orientation changes in a pH-dependent way in the apo form but not in the holo. This provides a possible explanation of why some residues near the interface affect the pH-sensitivity of PLM binding.

Glu-89 appears to be involved in both monomeric and dimeric PLM binding free energy. This dual involvement suggests that Glu-89 strongly influences the binding, possibly due to its role in the Tanford transition (the opening/closing of the pocket entrance), which occurs near the pH region where the binding is most favorable.

### 3.3 Structural Characterization of Bound Palmitate

To characterize PLM configurations, free energy landscapes were obtained from PCA (see Section 2.6) that captured PLM’s conformation and position in the pocket, as shown in Figure 7.

**Figure 7:**
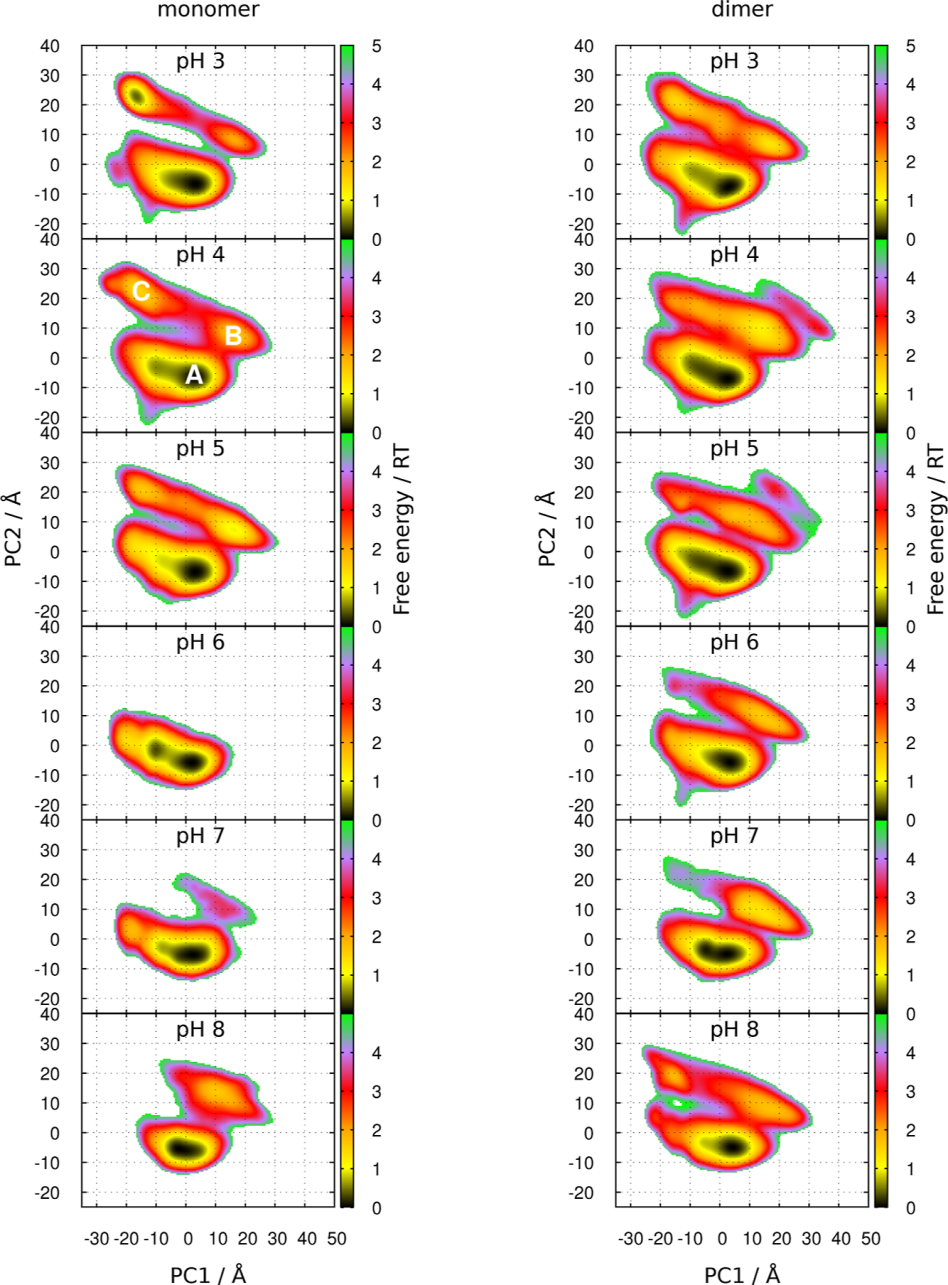
Free energy landscapes over the first two principal components (PC1 and PC2) obtained from the PCA of the PLM configurations, at different pH values. The location of basins A, B and C is shown in the landscape for pH 4.

Three main basins were identified, sometimes containing sub-basins. The first basin (basin A), roughly centered around (0, −5), has the majority of all PLM population and is present at all pHs. The other two basins, roughly centered around (15,5) (basin B) and (−15, 20) (basin C) are more prominent at pHs between 3 and 5. A high-energy basin is also present in the dimer at pH 4 and 5, adjacent to basin B. The absence of some of these basins or the less distinct patterns observed between pH 6 and 8 might be attributed to the protonation of PLM, which occurs at pH 5.7. To better understand the configurations associated with these basins, we measured the depth of PLM within the pocket and whether it is in an extended or more bent state; the mapping of these quantities on the landscapes is presented in Figures S2 and S3 of the Supporting Information. A visual representation of the configurations in each of the three main basins (A, B and C) is shown in Figure 8. In basin A a deeper and extended PLM is observed, while basins B and C display more surface-oriented configurations near the entrance of the pocket, but with the carboxyl head facing opposite directions. The configurations in basins B and C can be either extended or bent.

**Figure 8:**
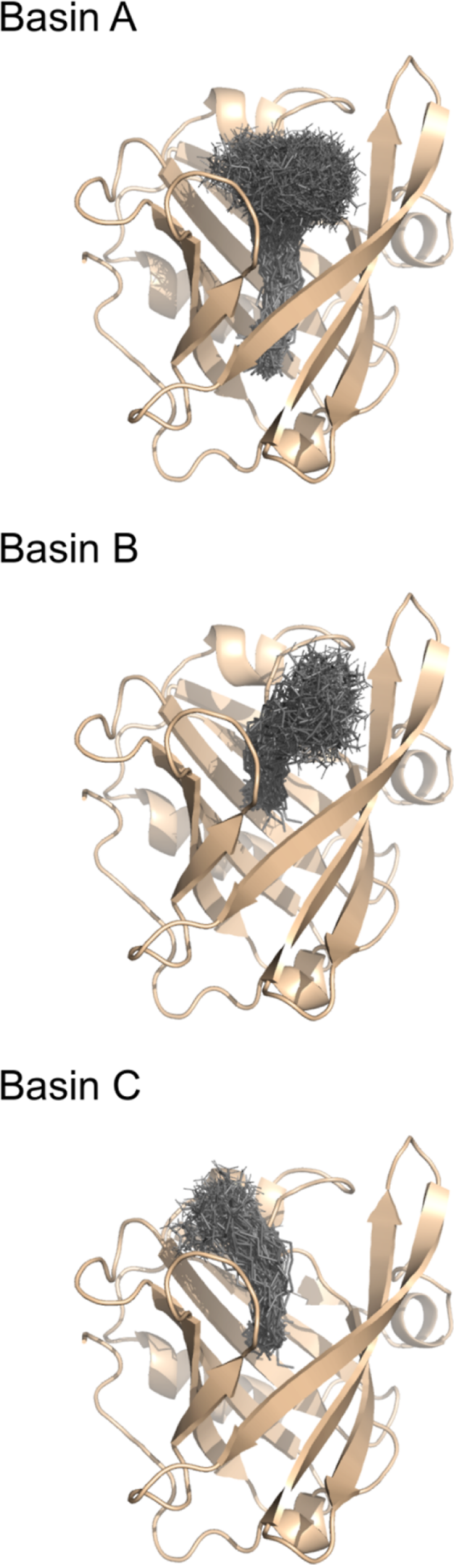
Representation of the three basins (A, B, and C). The monomer is depicted in dark yellow, while PLM structures are shown in gray. For each basin, the following procedure was adopted: (1) for each pH value, a set of all configurations within the deepest sub-basin of the corresponding region was selected; (2) 99% of the configurations within this set were randomly discarded to avoid too many frames; (3) the resulting sets corresponding to all pH values were collected; (4) this collection of frames is the one shown in the figure. Although only the monomer is shown here, the dimer follows the same pattern.

Some studies have suggested that Lys-60 and Lys-69 may be the residues that most influence the binding through close-by interactions.^53,54,95^ So, we measured the distance between these two lysines and the carboxylic head of PLM. Figure 9 shows that the distances are mainly within the range of 0.2–1.0 nm, indicating the potential formation of hydrogen or salt bonds, especially at high pH, when PLM becomes charged. It is also at high pH that PML binds more favorably (Figure 3) and tends to be more extended and deeply inserted in the pocket (Figure 7), suggesting that Lys-60 and Lys-69 may indeed help to stabilize the bounded ligand by keeping it “in place”.

**Figure 9:**
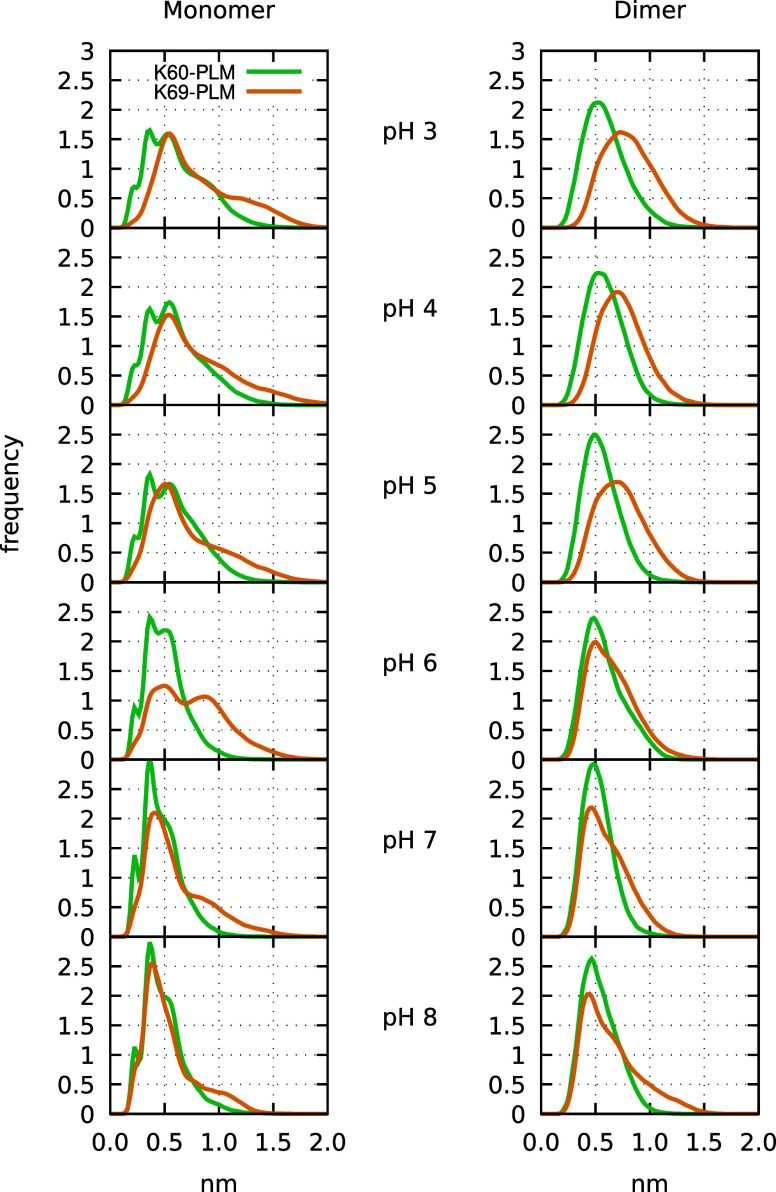
Histogram of the distance of Lys-60 and Lys-69 to PLM. This was calculated by measuring the distance between the amino nitrogen of the lysine side-chain and each of the two oxygen atoms of the carboxylic head of PLM, and selecting the smallest value.

### 3.4 Dimerization Free Energy

The relative dimerization free energy of the holo form was obtained using both the spline- and Hill-based methods. Since in this case no experimental data was available for the vertical fit, a reference absolute free energy at pH 7 was obtained from eq 1, using Δ*G*_bind_^M^ = −8.3 kcal/mol and Δ*G*_bind_^D^ = −14.5 kcal/mol from the experimental study of Wang et al.,^56^ and Δ*G*_dim_^apo^ = −7.16 kcal/mol from the spline-based calculations in ref 35 (obtained by fitting the computed profile to multiple experimental studies). The spline and Hill curves were then fitted to this reference value, yielding the absolute dimerization free energy of the holo form of BLG presented in Figure 10 (upper panel). The free energy of the apo form, which was calculated in ref 35, is also presented (lower panel). The dimerization of the holo form is favored the most around the pI, as previously observed for the apo form.^35,37^ As for the shape of the curve, it is similar to the one obtained for the apo form,^35^ suggesting that the binding of PLM does not impact the pH-sensitivity of the dimerization process. However, the dimerization of the apo form releases a higher amount of free energy, indicating that the absence of a bound ligand favors dimerization even more.

**Figure 10:**
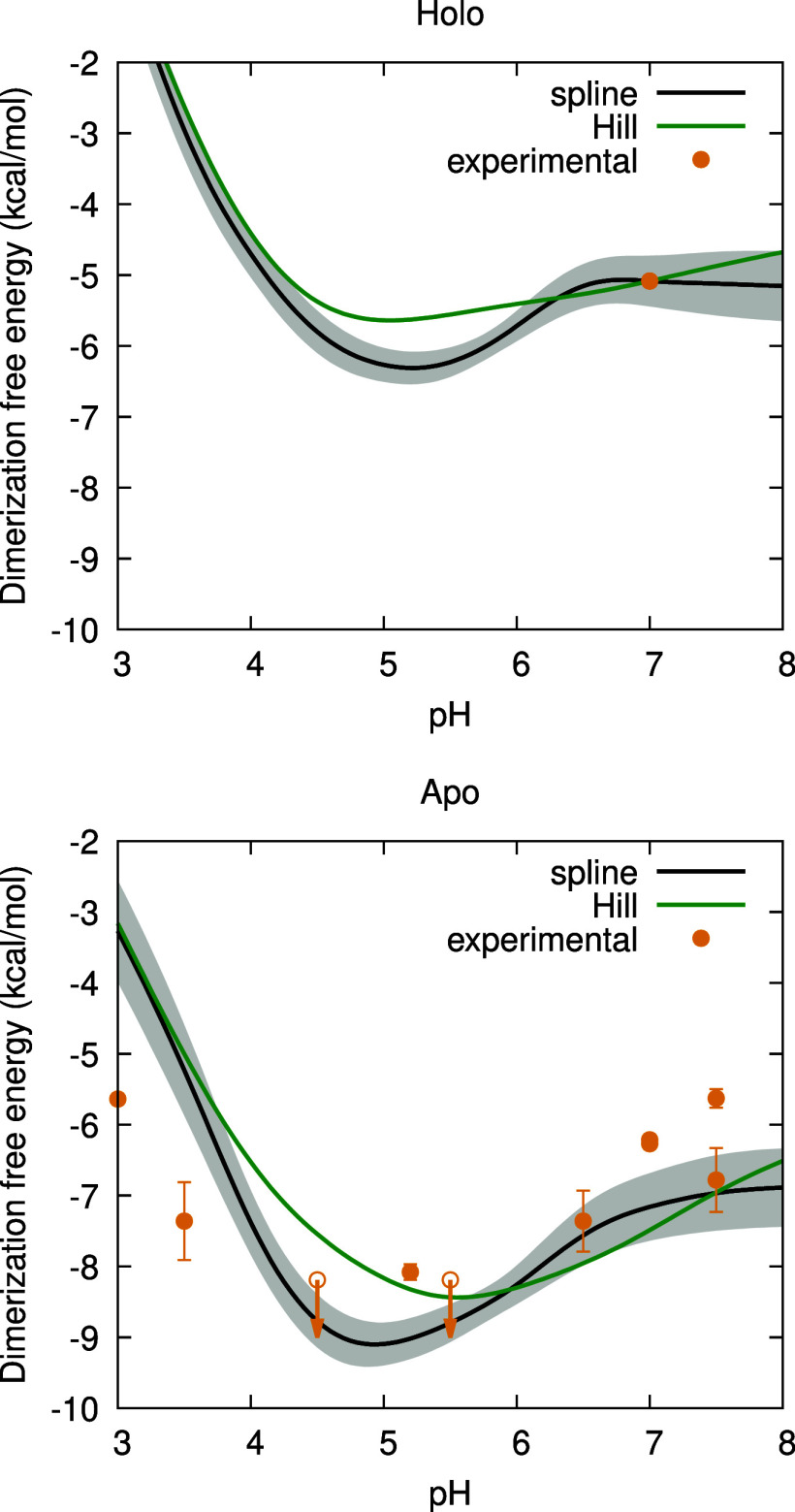
Dimerization free energy as a function of pH, calculated with the splines-based (black line) and the Hill-based (green line) methods. The gray shadow area delimits the error bounds obtained using a bootstrap method, as explained in Section 2.5. The upper panel depicts the dimerization free energy of the holo form fitted to the reference value Δ*G*_dim_^holo^(7) = −5.08 kcal/mol (yellow dot), the calculation of which is explained in the main text. The lower panel refers to the apo form (Reproduced from ref 35. Copyright 2022 American Chemical Society).

Regarding the individual residues, although the majority does not contribute substantially to the pH-dependency, some favor association or dissociation, depending on the pH (Figure 11). The residues that contribute the most are found at the interface, such as Asp-33, Asp-129, Asp-130, Glu-134, Asp-137, His-146 and His-161. As previously observed for the apo form,^35^ the contribution from His-161 is affected by substantial uncertainty, probably due to its very slow proton exchange rate during the CpHMD simulations (see Section 3.7).

**Figure 11:**
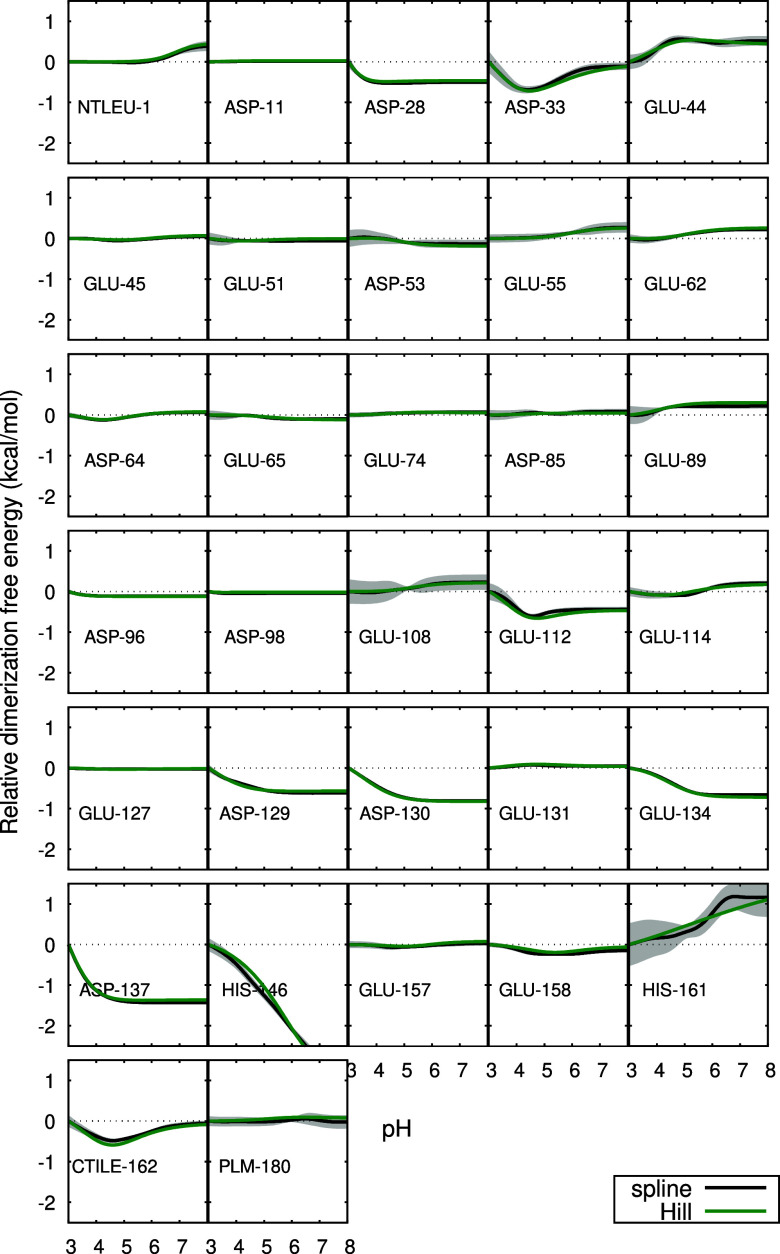
Site-specific contributions for the dimerization free energy relative to pH 3, as a function of pH. For further details, see the caption of Figure 10.

### 3.5 Allosteric Coupling

In order to quantitatively characterize the pH-dependent effect of ligand binding on dimerization, and vice versa, we extend the concept of coupling introduced by Weber.^96,97^ It was primarily introduced to quantify the reciprocal coupling effect that the binding of a ligand at one site has on the binding of a ligand at another site, but it can be extended to the binding–dimerization coupling addressed here. Thus, we define the corresponding allosteric coupling as

7 $$\Delta G_{coupl}=\Delta G_{\dim}^{holo}-\Delta G_{\dim}^{apo}=\Delta G_{bind}^{D}-2\Delta G_{bind}^{M}$$

The first difference expresses how dimerization is affected by the presence of the PLM ligand, while the second expresses how that binding is affected by the formation of a dimer, the two being necessarily identical (see Figure 1). Weber actually discusses the binding of two ligands to two associating monomers (Figure 5 in ref 96), as considered here, including the possibility of cooperativity between the two binding steps. However, since we do not address here the semiholo form of BLG, the Δ*G*_coupl_ just defined provides an adequate measure of the coupling between dimerization and the binding of two PLM molecules. Furthermore, we note that Δ*G*_coupl_ is also the free energy of the reaction

8 $$2ML+D\to {2M+DL}_{2}$$

So, the allosteric coupling also measures how favorable it is for two ligands to move from two monomers to one dimer, providing an alternative interpretation of allostery.

The pH-dependent allosteric coupling, computed from the free energy splines presented in the previous sections, is shown in Figure 12. An antagonistic relationship between dimerization and PLM binding is present, as Δ*G*_coupl_ > 0 (unfavorable) at all pHs; the binding of PLM disfavors dimerization and, simultaneously, dimerization disfavors the binding of PLM. A similar, although smaller, antagonistic effect was experimentally observed at pH 7 when using dodecyl sulfate as a ligand to BLG (at ionic strength of 0.1 M and 25 °C): from Table 1 of ref 98, we get

$$\Delta G_{coupl}=\Delta G_{\dim}^{holo}-\Delta G_{\dim}^{apo}=+0.9kcal/mol$$

Therefore, this antagonistic effect might be a general feature of negatively charged amphiphilic ligands of BLG. Given the incomplete knowledge of the biological function of BLG in ruminants, it is difficult to speculate on the biological relevance of this antagonistic effect. It might be related with regulatory aspects, possibly involving also binding cooperativity, which was experimentally observed in the binding of dodecyl sulfate^98^ (the computational study of cooperativity in BLG would require simulating the single-occupied dimer, not done in the present work). This type of behavior was also observed in hemoglobin, which besides an antagonistic effect involving the binding of the subunits and the binding of oxygen or carbon monoxide, also exhibits ligand binding cooperativity.^99^

**Figure 12:**
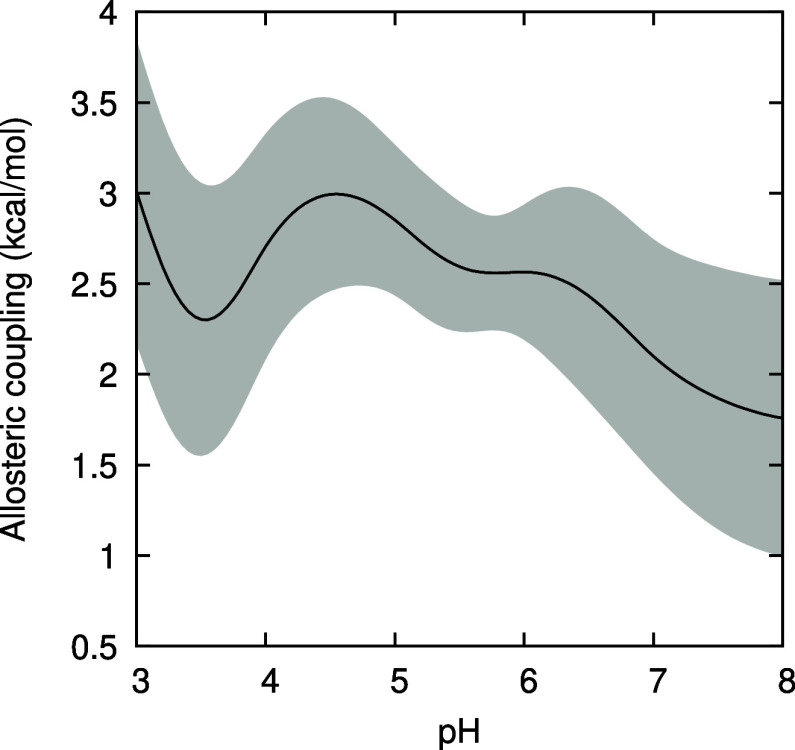
Allosteric coupling between dimerization and PLM binding, obtained from the free energy splines. The gray shadow area delimits the error bounds obtained using a bootstrap method.

**Table 1:** Pairs of Sites with an Absolute Protonation Correlation Greater Than 0.2

residue pair	pH	correlation
monomer		
Glu-44 – Glu-45	5	–0.20(1)
Glu-44 – His-161	3	–0.23(3)
Glu-44 – His-161	4	–0.31(4)
Glu-44 – His-161	5	–0.20(7)
Glu-44 – His-161	6	0.23(16)
Glu-127 – Asp-129	4	–0.26(1)
dimer, same chain		
Glu-108 – Glu-112	4	–0.20(3)
Glu-112 – Glu-114	4	–0.26(4)
Glu-127 – Asp-129	4	–0.24(1)
Asp-129 – Asp-130	3	–0.23(2)
dimer, different chains		
Asp-33 – Asp-33	4	–0.32(14)
Asp-33 – Asp-33	5	–0.22(7)
Asp-33 – His-161	3	–0.26(6)
Asp-33 – His-161	5	–0.24(6)
His-146 – His-146	7	0.31(17)
His-146 – His-161	5	–0.26(6)
His-146 – His-161	6	–0.24(8)
His-146 – His-161	7	–0.26(9)
His-161 – His-161	7	0.26(11)

### 3.6 Dimer Configuration

In our study of the apo form,^35^ we have identified two predominant dimer configurations: a “compact state” where the two monomers are in closer proximity (observed at pH 3 and 4), and a “relaxed state” where the monomers are not as close and exhibit a relative rotation (observed at pH 4–8). These configurations were detected through analyses of the contact surface area and the center-of-mass distance between the two dimer partners, as well as the dihedral angles between secondary-structure elements at the dimer interface. To compare these findings with the holo form, we conducted similar analyses.

The interpartner distance and contact surface area are depicted in Figure 13. The distribution of the interpartner distance is very similar at all pH values, showing no indication of any compaction. Regarding the contact surface analysis, we observed some higher-contact structures at pH 3 and 4, but that population is quite small and in sharp contrast with the clear two-peak transition previously observed in the apo form.^35^

**Figure 13:**
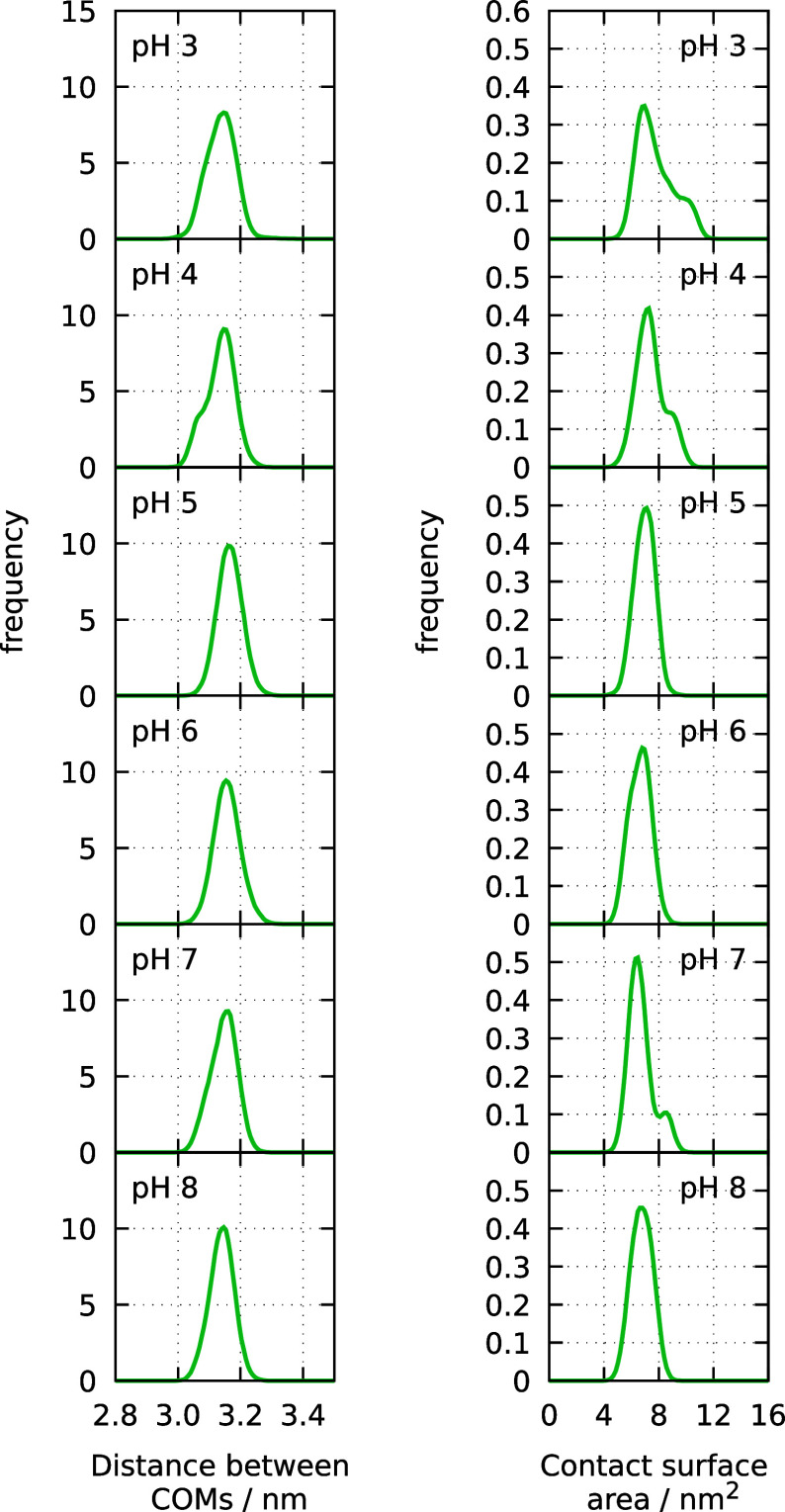
Histograms of the distance between the centers of mass of the two dimer partners (left panel) and of the contact surface area between the two (right panel).

The relative rotation of the two dimer partners was analyzed by measuring the dihedral angle between the two short β-strands and the two α-helices located at the interface (Figure 14). The angles show substantially higher fluctuations at higher pH than for the apo form,^35^ especially for the β-strands. This increased angle variability indicates greater flexibility in angle rotation compared to the apo form, although the specific transition observed in the apo form (deviation from the antiparallel alignment at low pH) is absent. Overall, these analyses seem to indicate that the binding of PLM induces the loss of the compact–relax transition previously observed for the apo form.

**Figure 14:**
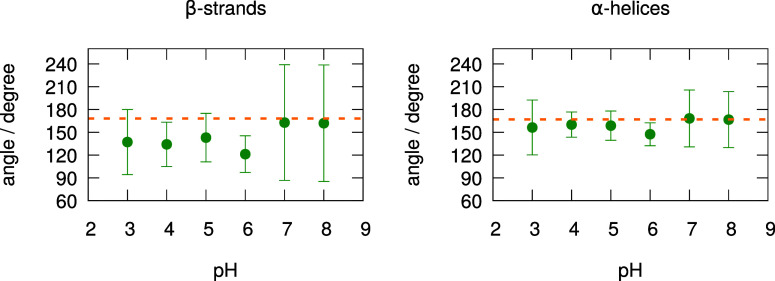
Dihedral angles between the two nonbarrel β-strands (left) and the two α-helices (right) in the dimer interface. The points and error bars were calculated as, respectively, the means and standard deviations of the angle average of each of the 8 replicates. The dihedral angles were defined using main chain atoms from the two opposite ends of each secondary structure motif, namely N-Ile-147_A_ →C-Ser-150_A_ →C-Ser-150_B_ →N-Ile-147_B_ for the β-strands and C-Ala-132_A_ →C-Leu-140_A_ →C-Leu-140_B_ →C-Ala-132_B_ for the α-helices. The dashed lines represent the average dihedral angles of the crystallographic structure used in this work (1b0O was obtained at pH 7.5 ^53^).

In the previous study of the apo form^35^ we found at pH 5, where dimerization is higher, a substantial charge complementarity between the two monomer surfaces that would be paired at the dimer interface. Thus, we suggested that this charge complementarity could favor dimerization. We now performed this same analysis for the holo form, using the distribution of sodium and chloride ions as a direct fingerprint of the protein electrostatics, since the ionic distribution reflects the charge distribution of the protein. The ionic distributions are presented in Figure 15, revealing a lower electrostatic complementarity than the one found for the apo form (see Figure 9 in ref 35). This may result in the higher mobility between the two partners (observed in the angle analysis) and lead to a reduced electrostatic attraction, thus explaining the less favorable dimerization.

**Figure 15:**
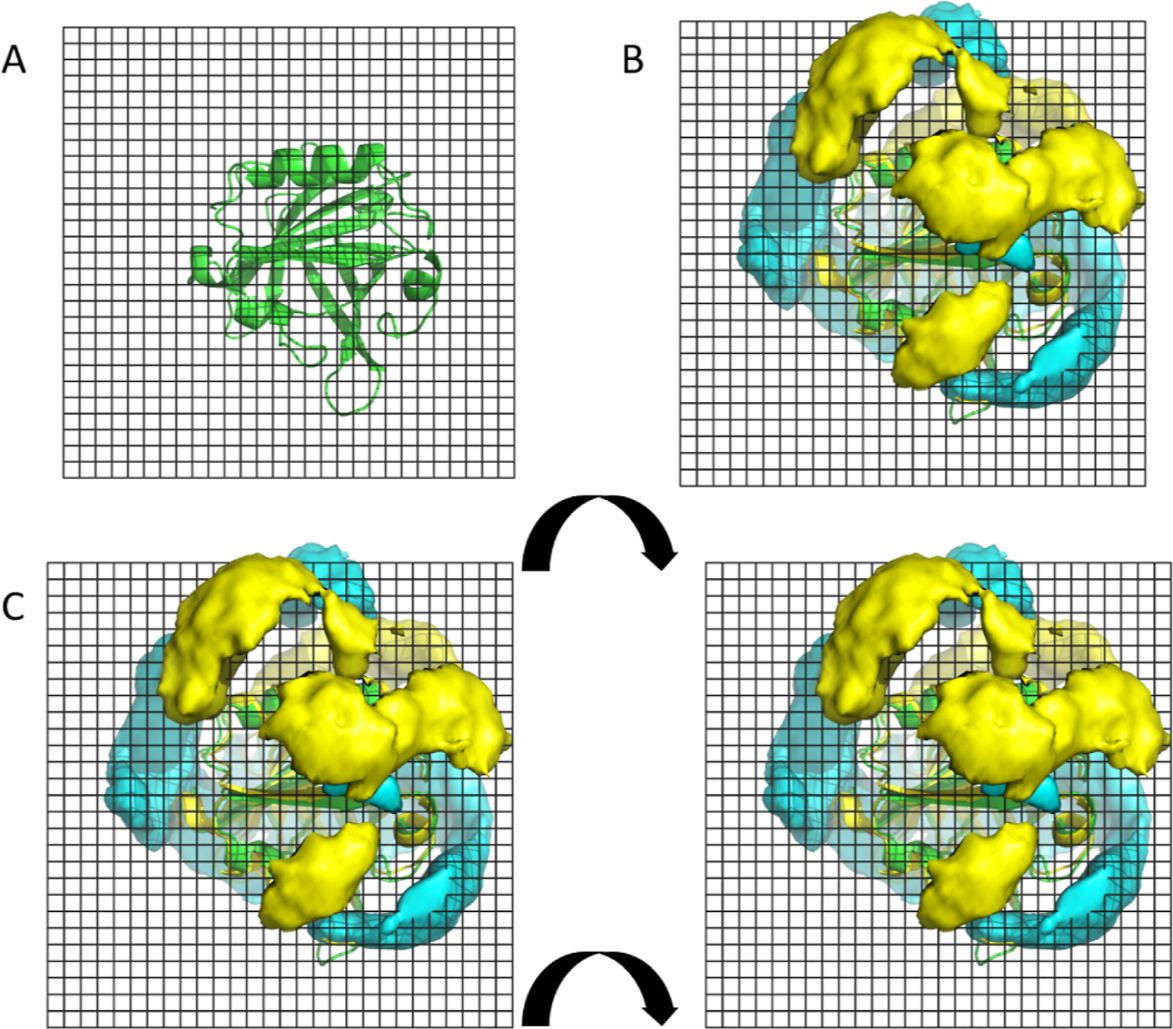
Dimer interface at pH 5. (A) Monomer face that would be found at the interface of the dimer, with the plane between both partners shown as a grid. (B) Density contours of 150 mM for Na^+^ (cyan) and Cl^–^ (yellow) ions computed from the monomer MD simulations at pH 5. (C) Interface of two potentially dimerizing partners. The two images are arranged in analogy to the facing pages of an open book; when the book closes, the two faces meet in the correct antiparallel configuration.

### 3.7 Protonation Correlations

It can be helpful to obtain protonation correlations among all sites to identify functional residues that are involved in electrostatics-dependent mechanisms.^73,100–104^ The pairs of sites in holo BLG that have an absolute correlation greater than 0.2 are listed in Table 1, together with the correlation value (with error) and pH. The sets of correlations in both the monomer and dimer, within the same chain and between different chains, are presented.

In the dimer, the residues pairs that have the higher correlations are primarily located at the dimer’s interface. These residues are also the ones that contribute the most to the pH-dependency of the dimerization free energy. This relationship was already observed in the apo form,^35^ where several strongly correlated protonation pairs were present and seemed to be related to the pH dependency of dimerization. These residues, predominantly slow proton exchangers (Figure S5 in Supporting Information), are even slower by around 2.5 times than those in the apo form. On the other hand, these strong correlations do not involve residues that contribute to the pH-dependency of the binding free energy.

In the monomer, only three strong correlated pairs were observed. Among these, only His-161, located at the interface, is a slow proton exchanger (Figure S4 in Supporting Information). Additionally, these residues do not seem to be the ones that contribute the most for the pH-dependency in the binding free energy.

Slow proton-exchanging sites might raise some concern about sampling, not only of protonation events, but also of the associated conformational changes. Indeed, slow conformational responses to the (de)­protonation of deeply buried sites have been previously observed,^105^ and this may lead some researchers to understandably avoid CpHMD simulations when studying large systems with multiple buried sites.^106,107^ Still, numerous protonation–conformation couplings have been successfully studied using CpHMD simulations (e.g., see ref 108 for recent examples), and good sampling strategies naturally depend on each methodological flavor. In the stochastic method used here, combining MM/MD and PB/MC, the use of multiple replicates seems to be a good strategy due to its ability to quickly give rise to a large diversity of transition regimes for sites exhibiting slow proton exchange (even by merely selecting different initial velocities). In the present study of holo BLG, His-161 is the slowest proton exchanger, and exhibits markedly different transition regimes in different replicates, as illustrated in Figure S6 for the dimer at pH 5 and 6. Therefore, as this case suggests, the use of multiple simulations might even be a better sampling alternative than the use of fewer and longer simulations. It should be noted, though, that His-161 is only transiently and mildly buried (see Figure 6) when compared to the extreme cases studied in ref 105; in such cases, longer simulations are most likely necessary.

When comparing the apo and holo forms, we observe fewer strong correlations in the latter, suggesting that the binding of palmitate decreases the reciprocal protonation effects between titratable sites. Nonetheless, it must be kept in mind that correlations are a ratio of second-order statistical moments and, as such, may take more time to converge than, say, mean values. Therefore, given that the simulation time used here for the holo form is twice that in the apo study, some of the strong correlations previously observed in the apo might have been spurious.

## 4 Conclusions

The present study provides insight on the allosteric mechanism between the binding of palmitate and the dimerization process in BLG. A full description of this interplay, characterized by their respective free energies, is obtained through a thermodynamic cycle (Figure 1) involving four pH-dependent free energy profiles. These profiles are computed from a differential linkage relation integrated using a thermodynamically based spline, with the absolute values being obtained by reference to experimental data. While Δ*G*_dim_^apo^(pH) has been previously determined in our study of the dimerization of the apo form, Δ*G*_bind_^M^(pH) and Δ*G*_bind_^D^(pH) are calculated using the reference data provided by Wang et al.^56^ The remaining one, Δ*G*_dim_^holo^(pH), although lacking experimental data, follows directly from the thermodynamic cycle. Another approach would be, instead of relying on experimental reference values for Δ*G*, to get them (at fixed protonation state) using absolute free energy calculations,^27,28,32^ but these are computationally heavier.

The binding free energy profiles show that binding is more favored between pH 6–7 for both the monomer and dimer, reflecting the need for the gate opening near that pH (Tanford transition).^49,50^ Despite this similar trend, the binding in the monomer is more favorable. It is interesting to note that the residues that contribute the most to the binding pH-dependency in the dimer are located at the interface, suggesting that the dimeric structure directly affects the pH-sensitivity of PLM binding through its interface. Glu89 also contributes substantially to this dependency in both the monomer and dimer, which can be related to its role in the Tanford transition.

The conformation and positioning of PLM within the pocket is analyzed using PCA-based free energy landscapes. Three main basins are identified, with the most populated one corresponding to an extended PLM molecule deeply inserted within the pocket. The other two basins correspond to more surface-oriented configurations near the entrance of the pocket that can be either extended or bent.

The dimerization of the holo form is most favored around the pI, consistent with observations made for the apo form^35,37–43^ and suggesting that the binding of PLM does not impact the pH at which the dimerization is more likely. However, the absence of a bound ligand appears to further enhance dimerization. When looking at the individual residues, the ones that contribute the most to the pH dependency of the dimerization free energy are located at the interface. In addition, we have computed the pH-dependent allosteric coupling, observing an evident antagonist relationship, where the binding of PLM disfavors dimerization and, conversely, dimerization disfavors the binding of PLM.

Analysis of the dimer configuration of the holo form has revealed a greater flexibility in the rotation between the two dimer partners compared to the apo form,^35^ and a loss of a previously observed compact state at low pH. Another striking difference from the apo form involves the loss of the charge complementarity previously observed at the interface, which seems to result from the higher mobility between chains. Since charge complementarity could assist in dimerization, this could explain the less favorable dimerization in the holo form.

Some pairs of titratable sites display correlated protonations in the dimer, being also the ones that contribute the most to the pH-dependency of the dimerization free energy. This relationship, previously observed in the apo form,^35^ is further emphasized. No such obvious relationship is observed for the monomer.

Overall, this study presents a general route to characterize the interplay between protein dimerization and ligand binding in a pH-dependent way, which can be easily extended to other combinations of processes. In particular, the calculation of a pH-dependent free energy profile for the allosteric coupling makes possible to easily identify the pH regions where the processes being studied exert an agonistic or antagonistic effect on each other. In the present application, the dimerization of BLG and its binding of palmitate are found to be antagonistic over the studied pH range. This might be a general feature of the binding of negatively charged amphiphilic ligands to BLG,^98^ but further studies are needed to clarify this.

## Supplementary Material



## Notes

Choice of reference free energy; p*K*_a_ values and titration curves; palmitate binding depth and head-to-tail length projected on the two first principal components obtained from analysis of binding configurations; protonation correlations; protonation transitions of His-161 (PDF)
