# Supplementary material for: Computing the pH-Dependent Thermodynamics of the Allostery between Dimerization and Palmitate Binding in β‑Lactoglobulin
Source: J Phys Chem B. 2025 May 23;129(22):5423–37. doi: 10.1021/acs.jpcb.5c01119 (PMC12820971; doi:10.1021/acs.jpcb.5c01119)
Supplement: Supplementary file 1 [file jp5c01119_si_001.pdf]

Supporting information for: Computing the pH-Dependent Thermodynamics of the Allostery between Dimerization and Palmitate Binding in β-Lactoglobulin

Lucie da Rocha, Sara R. R. Campos,* and António M. Baptista*

Instituto de Tecnologia Química e Biológica António Xavier, Universidade Nova de Lisboa, Av. da República, 2780-157 Oeiras, Portugal

E-mail: scampos@itqb.unl.pt; baptista@itqb.unl.pt

Phone: +351-214469619

Contents

List of Tables **S2**

List of Figures **S2**

1 Choice of reference free energy **S4**

2 Protonation **S6**

3 Structural characterization of bound palmitate **S11**

4 Protonation Correlations **S13**

References **S16**

List of Tables

S1 pK_a values in the monomeric and dimeric forms of apo and holo BLG, along with the corresponding differences for dimerization and binding. S6

List of Figures

S1 Average protonation of holo BLG sites in the monomer (purple) and dimer (green), showing the pK_a values and Hill coefficients h obtained from the fit of a Hill curve. S7

S2 Average distance between the last aliphatic carbon of PLM (tail end) and the bottom of the pocket (gamma-carbon of Leu-103) mapped onto the projection space of the PCA of Figure 7. . . . . S11

S3 Average distance between the first and last carbon atoms of the PLM chain, mapped onto the projection space of the PCA of Figure 7. . . . . S12

S4 Correlation time of the proton occupancy (0 or 1) of the BLG sites in the holo monomer, computed as the time at which the protonation autocorrelation function becomes lower than 0.1. . . . . S13
S5 Correlation time of the proton occupancy (0 or 1) of the BLG sites in the holo dimer, computed as the time at which the protonation autocorrelation function becomes lower than 0.1. . . . . S14
S6 Protonation transition events for His-161 in the eight replicate simulations (r1–r8) of the BLG dimer at pH 5 and 6. Lower/upper levels correspond to deprotonated/protonated. S15

1 Choice of reference free energy

As pointed in section 2.5, the pH-dependent free energy profiles obtained from the linkage relation are defined up to a constant. In our previous study of the apo dimerization,[S1] the required vertical shift of the profile was done by means of a least-squares fit to experimental data from several studies of the same BLG variant at the same ionic strength and temperature conditions, and covering the pH range of interest. The situation turns out to be more problematic with regard to palmitate binding. Although binding free energies have been determined from various experimental studies,[S2–S11] they are far from being a suitable set, for several reasons. First, none of these articles directly reports the ionic strength, and its calculation from the provided buffer details[S12] yields quite low values of 10–50 mM for some of the studies.[S5,S8–S11] Additionally, these studies were conducted under different or unspecified experimental conditions, rendering them unsuitable for a reliable comparison with our simulations. An exception was found in the work of Wang et al.,[S6] where the experimental conditions matched ours (temperature of 296 K and buffer-derived ionic strength of 0.107 M). Although the variant is not indicated, BLG seems to have been prepared from normal milk (i.e., from non-homozygote cows), which would typically contain around 1.5 times more variant A than B.[S13] Studies with other ligands seem to indicate that the binding differences between the A and B variants are very small, with B only slightly favoring the binding of lauric acid and sodium dodecyl sulfate by, respectively, 0.4 and 1.0 kcal/mol.[S14] Thus, the binding data from Wang et al. should provide an excellent approximation to those of variant A. Furthermore, they determined the binding constant at various BLG concentrations, which substantially affects the monomer–dimer equilibrium (e.g., see figure S1 in ref S1), even though they did not reach concentrations where BLG was exclusively in the monomeric or dimeric form. We decided to consider the monomeric and dimeric forms as approximately represented by the reported BLG concentrations of respectively 1 μM and 200 μM, thus assigning $\Delta G^{\mathrm{M}}_{\mathrm{bind}} = -8.3$ kcal/mol and $\Delta G^{\mathrm{D}}_{\mathrm{bind}} = -14.5$ kcal/mol, obtained from the single-chain binding constants reported for those concentrations.[S6] However, since

these concentrations do not still correspond to pure forms, the actual difference between the $\Delta G^{\mathrm{M}}_{\mathrm{bind}}$ and $\Delta G^{\mathrm{D}}_{\mathrm{bind}}$ is expected to be slightly greater. These free energy values are reported in ref S6 for a strongly binding site, presumably the pocket formed by the BLG calyx, but weak binding is also reported to a putative second type of binding site. Like for other BLG ligands, such secondary binding sites for PLM have been sometimes proposed when interpreting binding data,[S2,S5,S6] but none were observed in the crystallographic structure with bound PLM.[S15] This putative weak binding of PLM to non-calyx sites may simply reflect non-specific association due to its low solubility, being ignored here. Nonetheless, the binding model used by Wang et al., which assumes multiple independent binding sites, obviously affects the equilibrium constants that they compute from the raw data of the ultrafiltration equilibrium experiments; in particular, their model rules out the possibility of studying cooperativity between the two calyx sites in the dimer.

2 Protonation

Table S1: pK_a values in the monomeric and dimeric forms of apo and holo BLG, along with the corresponding differences for dimerization and binding. The values shown for the apo form are sourced from ref S1.

Site	pK_a				ΔpK_a			
	Apo		Holo		Dimerization		Binding	
	Mon	Dim	Mon	Dim	Apo	Holo	Mon	Dim
NTLEU-1	6.9	7.0	6.8 ± 0.3	7.0 ± 0.6	0.2	0.2	0.0	−0.1
ASP-11	3.2	3.2	3.0 ± 0.4	3.0 ± 0.2	0.0	0.0	−0.2	−0.2
ASP-28	3.3	3.1	3.1 ± 1.4	2.2 ± 4.4	−0.2	−1.0	−0.2	−0.9
ASP-33	4.2	4.4	4.1 ± 1.2	3.5 ± 2.3	0.2	−0.5	−0.2	−0.9
GLU-44	4.1	4.2	4.2 ± 1.1	4.5 ± 0.3	0.1	0.3	0.1	0.3
GLU-45	5.5	5.5	5.3 ± 0.2	5.3 ± 0.3	0.0	0.0	−0.3	−0.3
GLU-51	3.7	3.6	3.9 ± 0.9	3.9 ± 0.5	−0.1	0.0	0.2	0.3
ASP-53	4.9	4.8	4.4 ± 1.1	4.4 ± 0.7	−0.1	−0.1	−0.5	−0.4
GLU-55	5.9	6.0	5.8 ± 1.0	5.9 ± 0.6	0.2	0.1	0.0	−0.1
GLU-62	4.4	4.3	4.4 ± 0.5	4.5 ± 0.5	−0.1	0.1	0.0	0.2
ASP-64	4.2	4.3	4.2 ± 0.2	4.2 ± 0.3	0.1	0.0	0.0	−0.1
GLU-65	4.7	4.7	4.7 ± 0.5	4.6 ± 0.4	0.0	0.0	−0.1	−0.1
GLU-74	4.9	4.8	5.2 ± 0.5	5.2 ± 0.2	0.0	0.0	0.3	0.3
ASP-85	4.1	3.9	4.3 ± 0.8	4.3 ± 0.3	−0.2	0.0	0.3	0.5
GLU-89	4.4	4.7	4.0 ± 1.4	4.1 ± 0.3	0.4	0.1	−0.4	−0.6
ASP-96	2.7	3.0	2.8 ± 0.7	2.7 ± 0.7	0.2	−0.1	0.1	−0.3
ASP-98	2.4	0.9	2.5 ± 9.2	1.8 ± 7.6	−1.4	−0.6	0.1	0.9
GLU-108	5.5	5.1	5.0 ± 2.0	5.1 ± 1.2	−0.4	0.1	−0.5	0.0
GLU-112	3.7	3.9	4.3 ± 1.0	4.0 ± 0.8	0.2	−0.3	0.6	0.2
GLU-114	4.3	4.3	4.3 ± 0.6	4.3 ± 0.5	0.0	0.0	0.0	0.0
GLU-127	4.3	4.1	4.4 ± 0.1	4.4 ± 0.1	−0.2	0.0	0.0	0.3
ASP-129	3.8	3.4	3.6 ± 0.5	3.2 ± 0.9	−0.4	−0.4	−0.1	−0.2
ASP-130	3.9	3.1	3.9 ± 0.2	3.4 ± 0.4	−0.8	−0.5	0.0	0.3
GLU-131	4.1	3.9	4.2 ± 0.3	4.3 ± 0.1	−0.1	0.0	0.1	0.3
GLU-134	4.7	4.6	4.7 ± 0.1	4.4 ± 0.2	−0.1	−0.3	0.0	−0.2
ASP-137	3.4	1.8	3.5 ± 0.3	1.7 ± 3.5	−1.6	−1.8	0.1	0.0
HIS-146	6.7	6.0	6.8 ± 0.6	5.6 ± 1.5	−0.6	−1.2	0.1	−0.4
GLU-157	5.3	5.4	5.3 ± 0.6	5.3 ± 0.2	0.1	0.0	0.0	−0.1
GLU-158	5.4	5.6	5.2 ± 0.3	5.2 ± 0.4	0.2	0.0	−0.2	−0.4
HIS-161	4.3	1.6	4.3 ± 6.2	5.2 ± 3.7	−2.8	0.9	0.0	3.6
CTILE-162	4.8	4.7	4.4 ± 0.3	4.2 ± 1.1	−0.1	−0.2	−0.4	−0.5
PLM	–	–	5.7 ± 1.1	5.8 ± 0.7	–	0.0	–	–

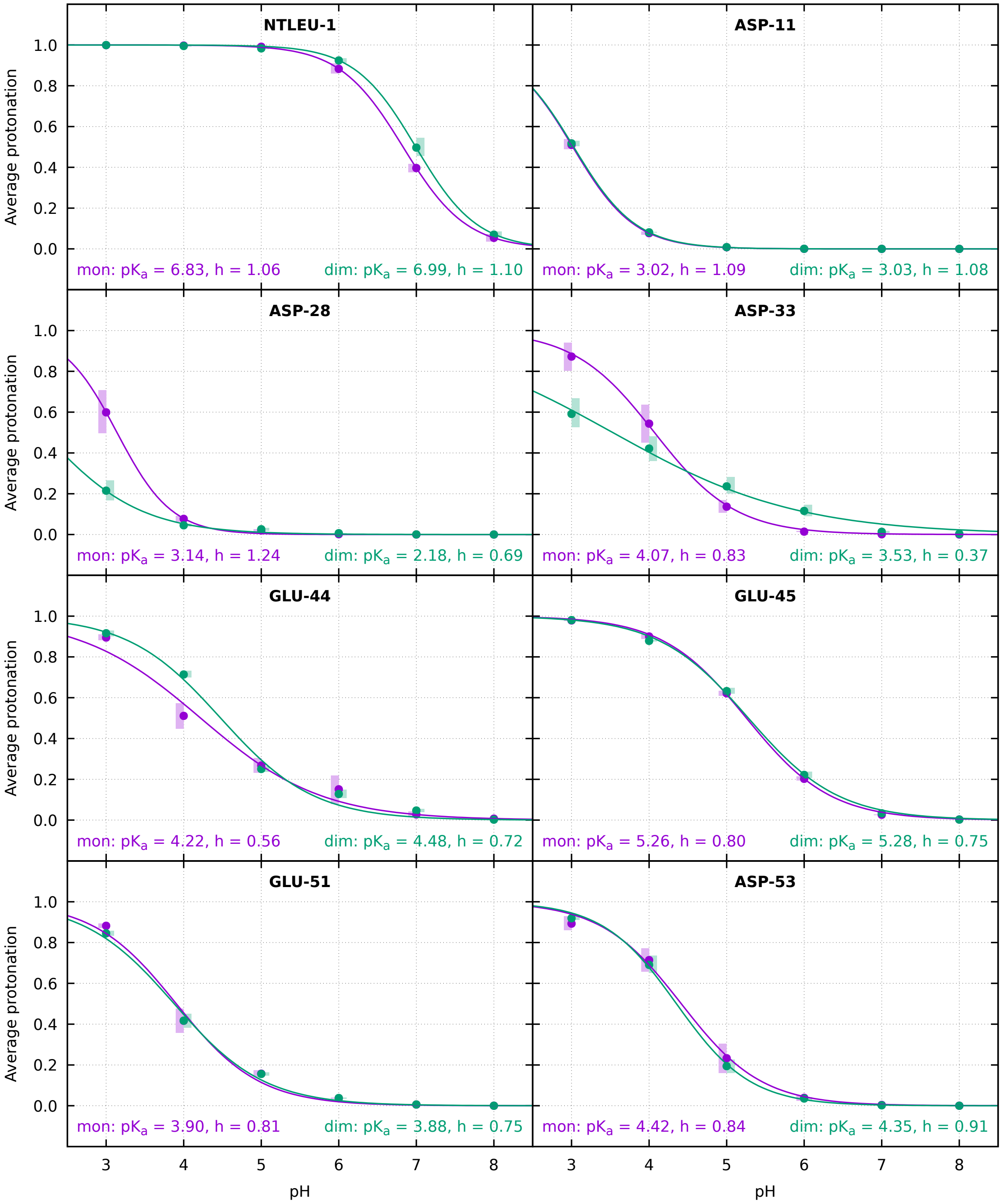

Figure S1: Average protonation of holo BLG sites in the monomer (purple) and dimer (green), showing the pK_a values and Hill coefficients h obtained from the fit of a Hill curve. Error bars are slightly displaced for easier visualization.

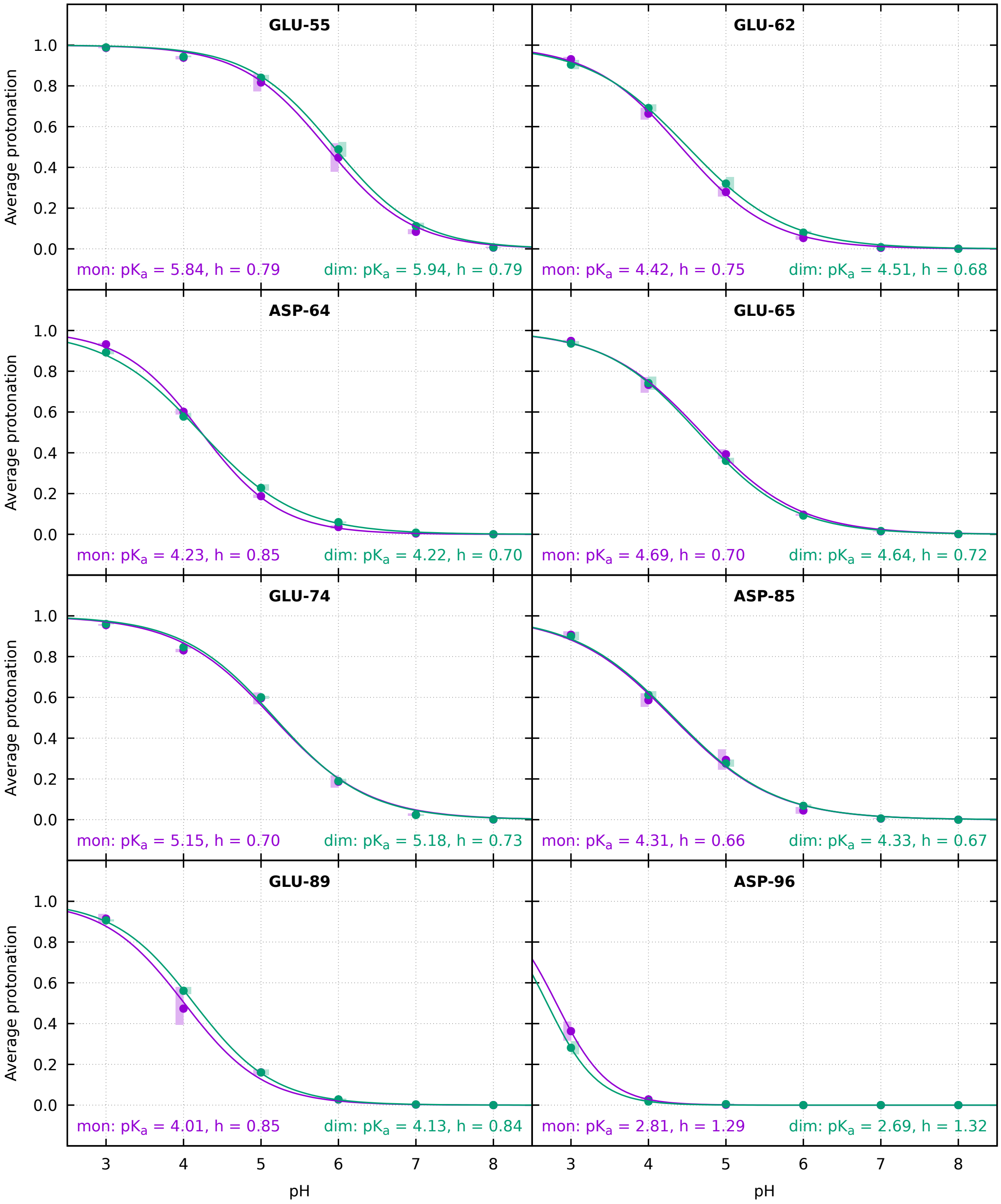

Figure S1: (continued, part 2)

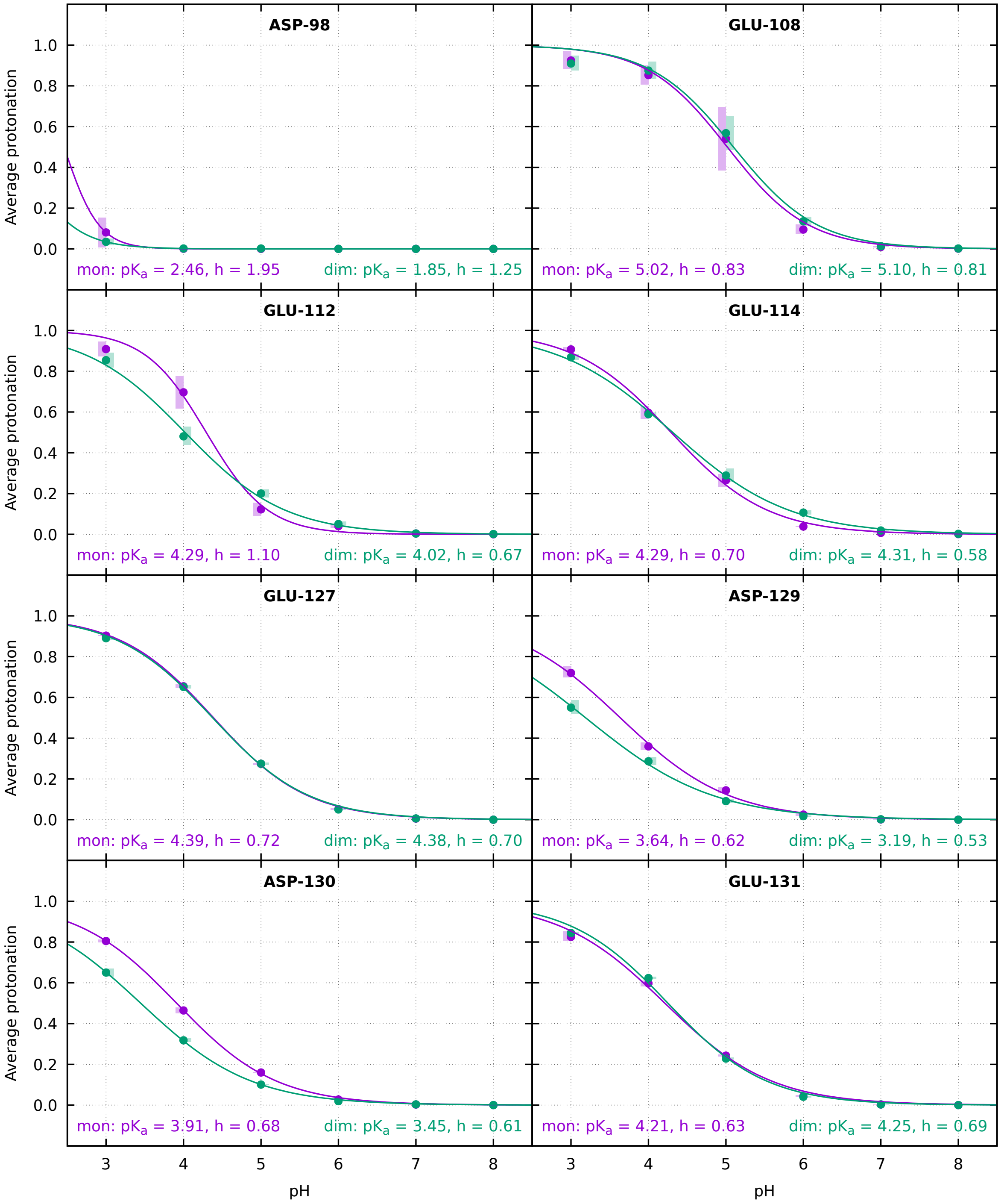

Figure S1: (continued, part 3)

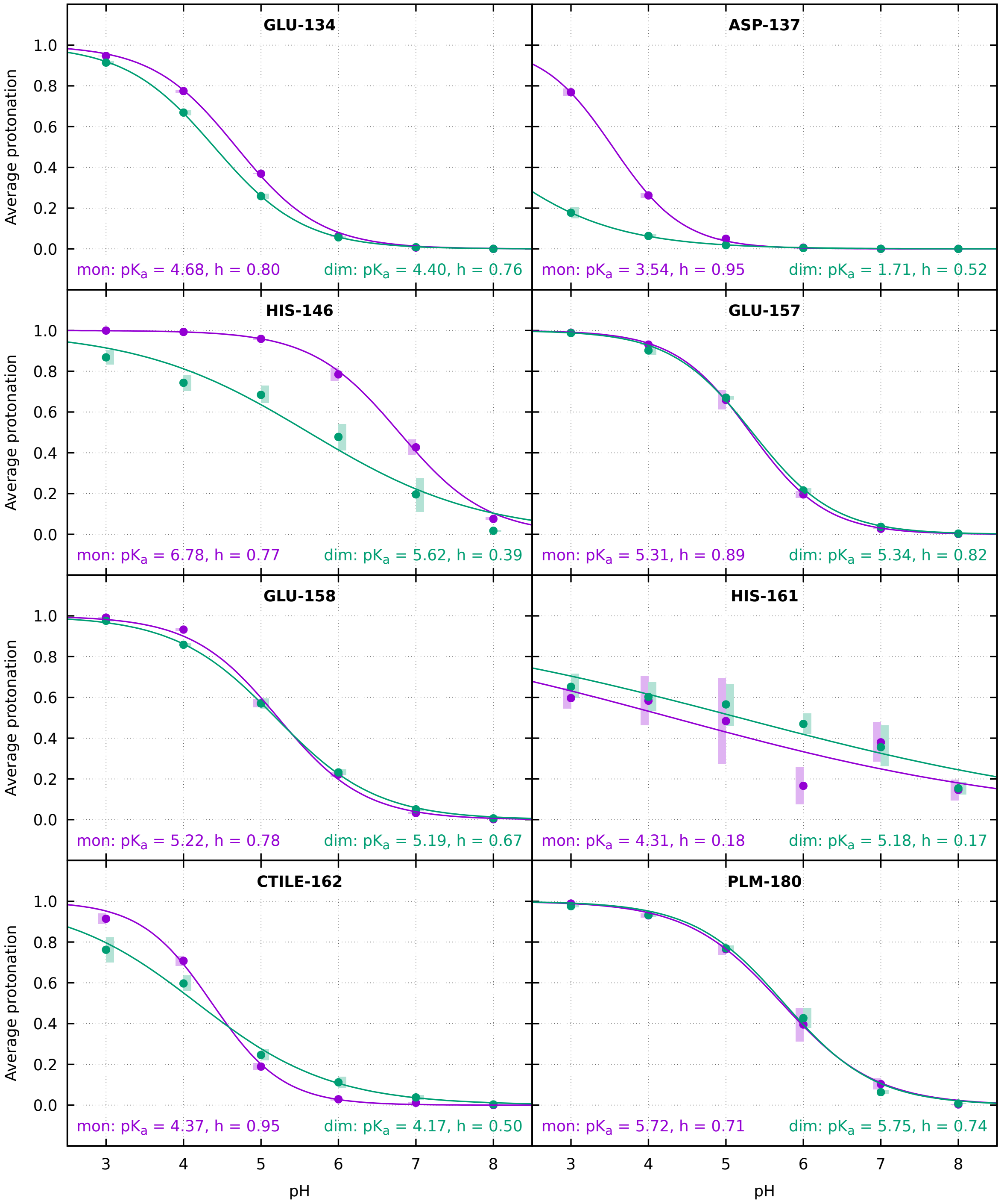

Figure S1: (continued, part 4)

3 Structural characterization of bound palmitate

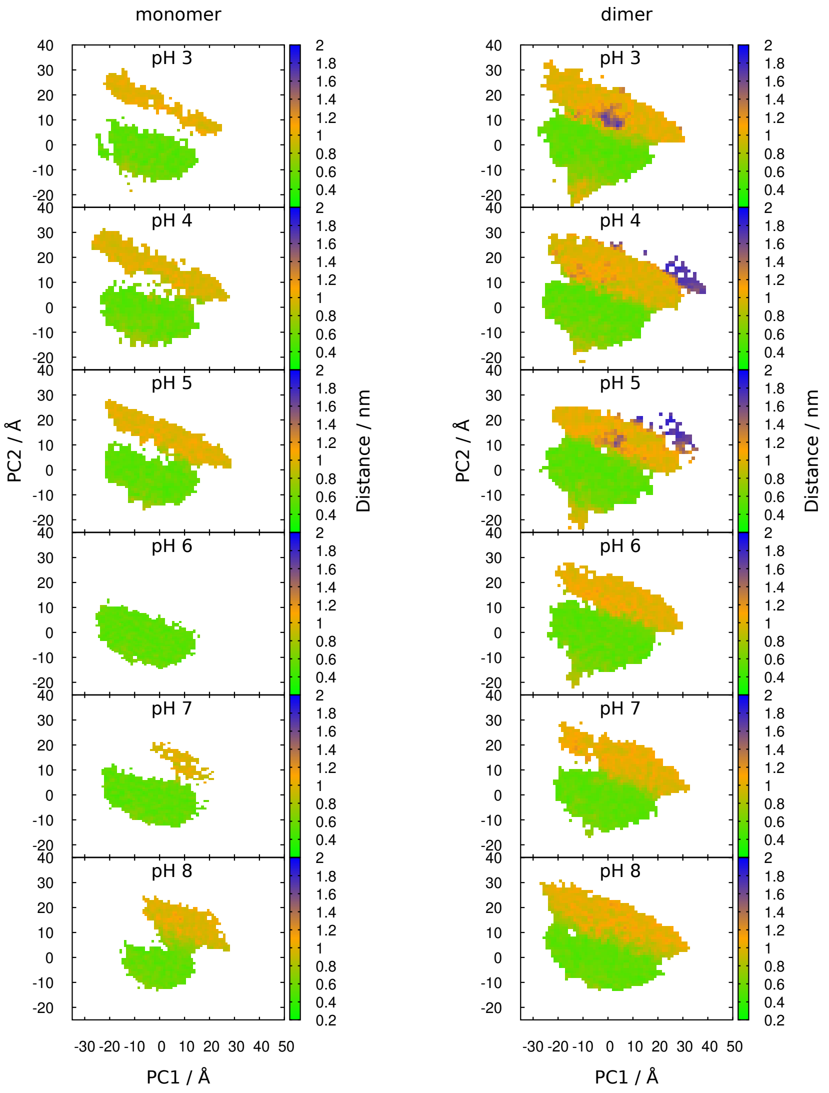

Figure S2: Average distance between the last aliphatic carbon of PLM (tail end) and the bottom of the pocket (gamma-carbon of Leu-103) mapped onto the projection space of the PCA of Figure 7.

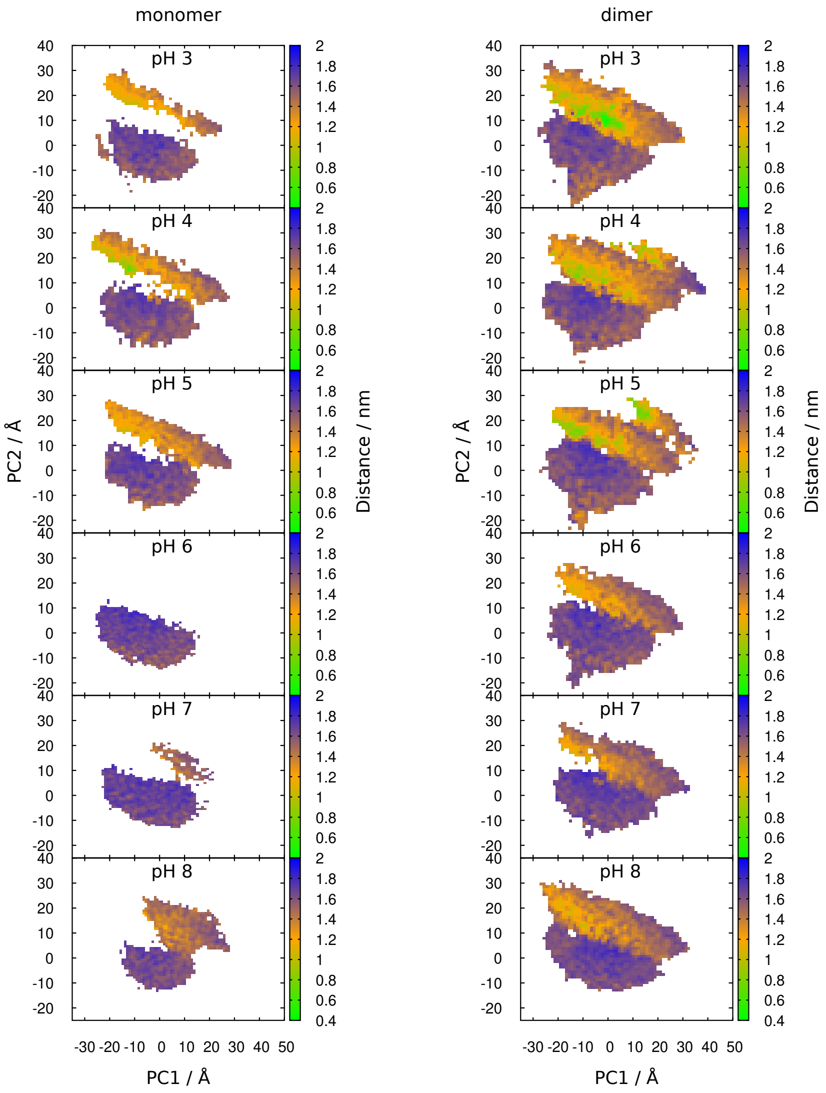

Figure S3: Average distance between the first and last carbon atoms of the PLM chain, mapped onto the projection space of the PCA of Figure 7.

4 Protonation Correlations

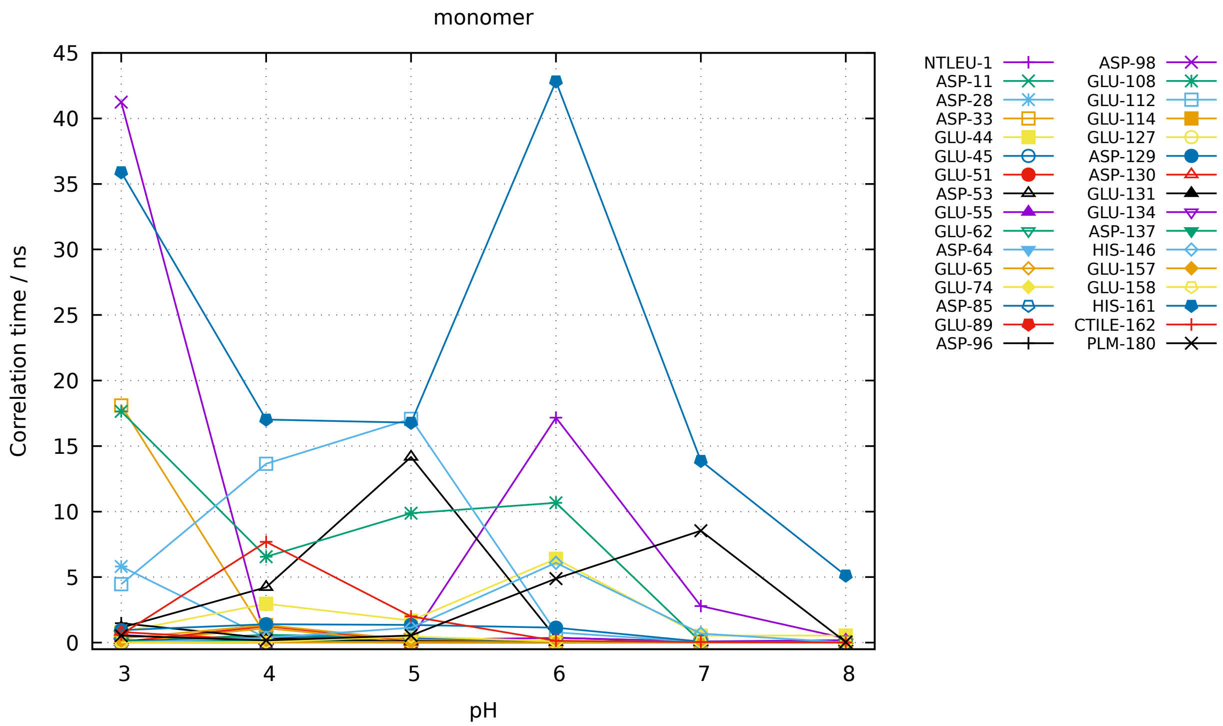

Figure S4: Correlation time of the proton occupancy (0 or 1) of the BLG sites in the holo monomer, computed as the time at which the protonation autocorrelation function becomes lower than 0.1.

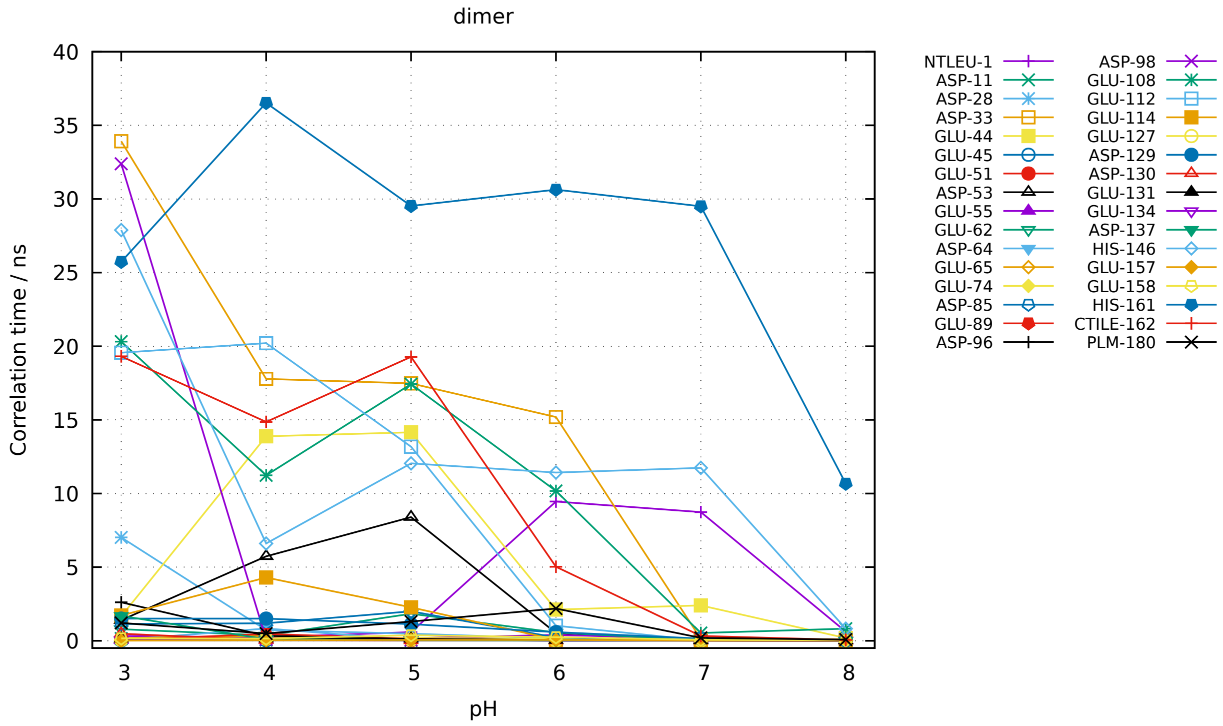

Figure S5: Correlation time of the proton occupancy (0 or 1) of the BLG sites in the holo dimer, computed as the time at which the protonation autocorrelation function becomes lower than 0.1.

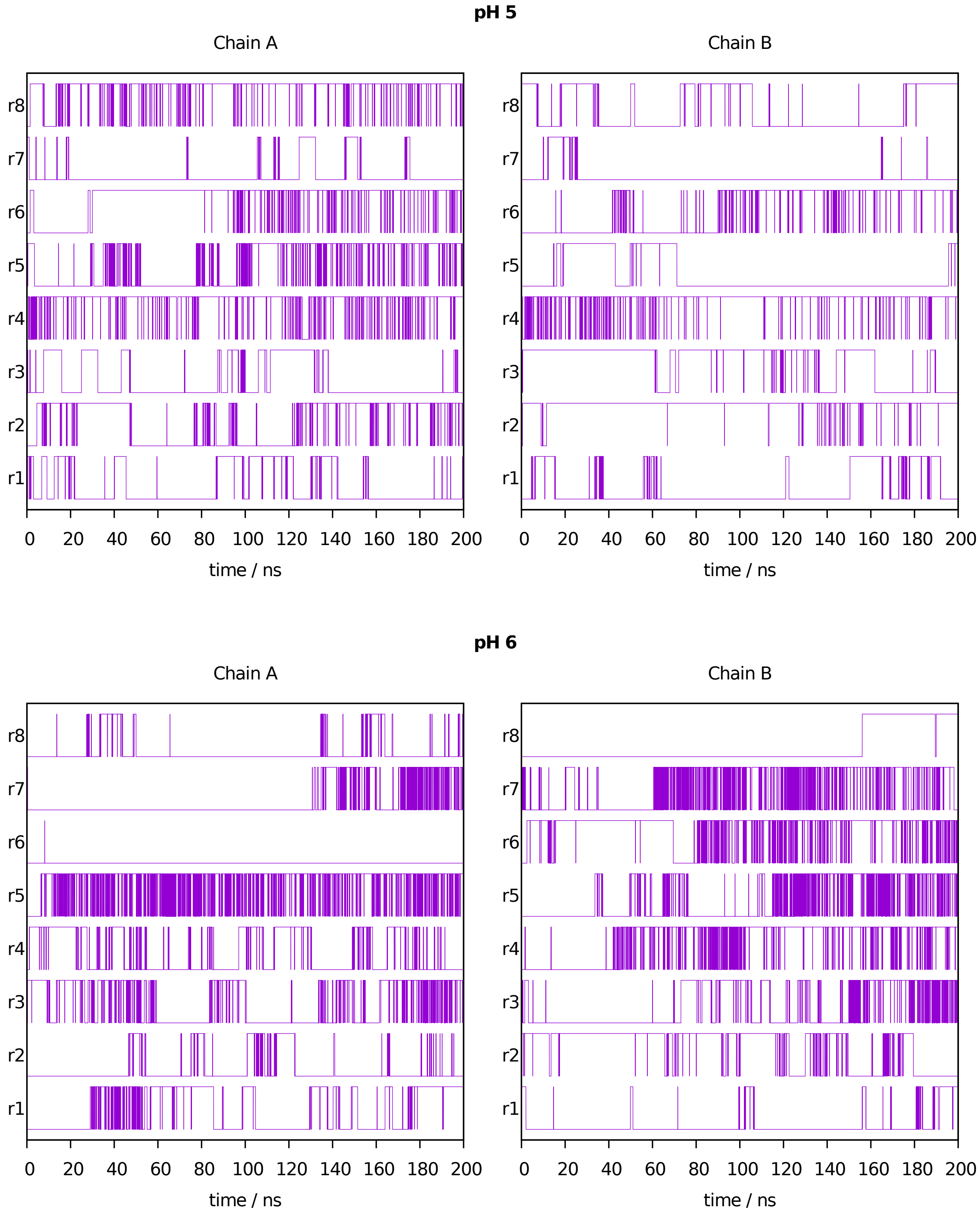

Figure S6: Protonation transition events for His-161 in the eight replicate simulations (r1–r8) of the BLG dimer at pH 5 and 6. Lower/upper levels correspond to deprotonated/protonated.

References

(S1) da Rocha, L.; Baptista, A. M.; Campos, S. R. R. Approach to Study pH-Dependent Protein Association Using Constant-pH Molecular Dynamics: Application to the Dimerization of β-Lactoglobulin. *J. Chem. Theory Comput.* **2022**, *18*, 1982–2001.

(S2) Spector, A. A.; Fletcher, J. E. Binding of long chain fatty acids to β-lactoglobulin. *Lipids* **1970**, *5*, 403–411.

(S3) Perez, M. D.; Sanchez, L.; Aranda, P.; Ena, J.; Oria, R.; Calvo, M. Effect of β-lactoglobulin on the activity of pregastric lipase. A possible role for this protein in ruminant milk. *Biochimica et Biophysica Acta (BBA)-Lipids and Lipid Metabolism* **1992**, *1123*, 151–155.

(S4) Frapin, D.; Dufour, E.; Haertle, T. Probing the fatty acid binding site of β-lactoglobulins. *Journal of Protein Chemistry* **1993**, *12*, 443–449.

(S5) Narayan, M.; Berliner, L. J. Mapping fatty acid binding to β-lactoglobulin: ligand binding is restricted by modification of Cys 121. *Protein Science* **1998**, *7*, 150–157.

(S6) Wang, Q.; Allen, J. C.; Swaisgood, H. E. Protein concentration dependence of palmitate binding to β-lactoglobulin. *Journal of Dairy Science* **1998**, *81*, 76–81.

(S7) Wang, Q.; Allen, J. C.; Swaisgood, H. E. Binding of lipophilic nutrients to β-lactoglobulin prepared by bioselective adsorption. *Journal of Dairy Science* **1999**, *82*, 257–264.

(S8) Collini, M.; D'Alfonso, L.; Molinari, H.; Ragona, L.; Catalano, M.; Baldini, G. Competitive binding of fatty acids and the fluorescent probe 1-8-anilinonaphthalene sulfonate to bovine β-lactoglobulin. *Protein Science* **2003**, *12*, 1596–1603.

(S9) Yang, M.-C.; Guan, H.-H.; Liu, M.-Y.; Lin, Y.-H.; Yang, J.-M.; Chen, W.-L.; Chen, C.-J.; Mao, S. J. Crystal structure of a secondary vitamin D_3 binding site of milk β-

lactoglobulin. *Proteins: Structure, Function, and Bioinformatics* **2008**, *71*, 1197–1210.

(S10) Loch, J. I.; Polit, A.; Bonarek, P.; Olszewska, D.; Kurpiewska, K.; Dziedzicka-Wasylewska, M.; Lewiński, K. Structural and thermodynamic studies of binding saturated fatty acids to bovine β-lactoglobulin. *International Journal of Biological Macromolecules* **2012**, *50*, 1095–1102.

(S11) Rovoli, M.; Thireou, T.; Choiset, Y.; Haertlé, T.; Sawyer, L.; Eliopoulos, E.; Kontopidis, G. Thermodynamic, crystallographic and computational studies of non-mammalian fatty acid binding to bovine β-Lactoglobulin. *International Journal of Biological Macromolecules* **2018**, *118*, 296–303.

(S12) Beynon, R. J.; Easterby, J. S. *Buffer Solutions*; Oxford University Press, Oxford: New York, 1996.

(S13) Graml, R.; Weiss, G.; Buchberger, J.; Pirchner, F. Different rates of synthesis of whey protein and casein by alleles of the β-lactoglobulin and α_{s1}-casein locus in cattle. *Genetics Selection Evolution* **1989**, *21*, 547–554.

(S14) Bello, M.; Portillo-Téllez, M. d. C.; García-Hernández, E. Energetics of ligand recognition and self-association of bovine β-lactoglobulin: differences between variants A and B. *Biochemistry* **2011**, *50*, 151–161.

(S15) Wu, S.-Y.; Perez, M. D.; Puyol, P.; Sawyer, L. β-Lactoglobulin binds palmitate within its central cavity. *Journal of Biological Chemistry* **1999**, *274*, 170–174.
